# Trends in global glucose lowering medication consumption: Insights from pharmaceutical sales data (2010–2021)

**DOI:** 10.1371/journal.pgph.0005326

**Published:** 2025-10-22

**Authors:** Myo Myo Swe Oo, Sahan Jayawardana, Allen Campbell, Murray Aitken, Kershaw V. Patel, Khuram Nasir, Mandeep Mehra, Elias Mossialos

**Affiliations:** 1 Department of Health Policy, London School of Economics and Political Science, London, United Kingdom; 2 IQVIA Institute for Human Data Science, Parsippany, New Jersey, United States of America; 3 Division of Cardiovascular Prevention and Wellness, Department of Cardiology, Houston Methodist DeBakey Heart & Vascular Center, Houston, Texas, United States of America; 4 Brigham and Women’s Hospital, Harvard Medical School, Boston, Massachusetts, United States of America; PLOS: Public Library of Science, UNITED STATES OF AMERICA

## Abstract

Diabetes imposes a substantial global burden. Examining consumption trends of glucose lowering medications can help facilitate cross-country comparisons and uncover areas of unmet need. Leveraging IQVIA MIDAS data, our analysis spans 72 countries and 2 regions from 2010 to 2021, employing defined daily dose (DDD) as a consumption metric. We assessed consumption trends across income tiers and individual country rankings, exploring WHO essential medicines, insulin, new drug classes and specific drugs, with further analyses on their correlation with treatment guideline releases. Global glucose lowering medication consumption rate increased from 39.2 to 54.0 DDD per thousand inhabitants per day (DDD/TID) between 2010 and 2021. Across the same period, median consumption rates were 60.1 DDD/TID [IQR, 46.5-70.6] in high-income countries (HICs), 26.9 DDD/TID [IQR, 8.0-51.3] in upper-middle income countries (UMICs) and 10.8 DDD/TID [IQR, 6.5-18.5] in low- and lower-middle income countries (LMICs). While the most significant consumption changes occurred in UMICs and LMICs such as Bosnia, China and Indonesia, HICs such as Finland, Canada and USA consistently showed the highest consumption rates. Over the study period, the median consumption rates for essential medicines, insulin and new drug classes increased, except for intermediate-acting insulin and soluble insulin/biosimilars. HICs drove the consumption of fast-acting and long-acting insulin and new drug classes, whereas UMICs and LMICs drove the consumption of intermediate-acting insulin. This study sheds light on the global variations in glucose lowering medication consumption, providing insights to address access gaps, particularly in UMICs and LMICs.

## Introduction

Diabetes mellitus affects 438 million people globally [1] with 90% of cases identified as Type-2 diabetes mellitus (T2DM) and the remaining as Type-1 diabetes mellitus (T1DM) [2]. Direct medical costs amount to 673 billion USD [3]. Patients with diabetes have a higher risk of developing cardiovascular complications, infections, vision loss, end-stage renal disease, and other comorbidities [4–6]. In 2019, 1.47 million deaths were attributable to T2DM, a 10.8% increase in age-standardised death rate (ASDR) compared to 1990 [1]. Among patients with diabetes, 80% reside in lower-middle-income countries (LMICs) [7] where T2DM related ASDR was at least three times higher than that in high-income countries (HICs) [8]. LMICs face significant economic burden, with diabetes costs representing 1.2% of cumulative GDP in sub-Saharan Africa, for example, and patients often experience financial strain from out-of-pocket treatment expenses [9], underscoring the critical need for equitable access to medication.

Management of T2DM usually involves lifestyle modification through diet and exercise, and medication. The medications used to treat T2DM have different mechanisms of action to lower blood sugar levels. Drugs belonging to different classes can be used as monotherapies or in combination with drugs from other classes to enhance glycaemic control. LMICs face significant challenges with treatment strategies due to limited medication accessibility, affordability issues and outdated clinical guidelines.

The medicines for the management of T2DM included in the WHO essential medicines list (EML) are metformin (500mg), soluble insulin (40 IU/ml, 100 IU/ml), and intermediate-acting insulin (40 IU/ml, 100 IU/ml), with gliclazide (30mg, 60mg, 80mg) added in 2013 [10]. Recently, the WHO added SGLT2 inhibitors such as empagliflozin, canagliflozin, and dapagliflozin, and long-acting insulin analogues and biosimilars such as insulin detemir, insulin degludec, and insulin glargine to the 2021 WHO EML [11]. Although insulin analogues were initially rejected for inclusion in the WHO EML in 2019 due to concerns over rising prices [12], their recent addition highlights the significance of improving access to these medicines through competitive pricing and biosimilar production. Long-acting insulin analogues were included due to their convenience and lower risk of hypoglycaemia compared to neutral protamine Hagedorn (NPH) insulin, while SGLT2 inhibitors were added based on their positive impact on patients with cardiovascular or renal diseases [11].

The WHO’s Global Action Plan for the Prevention and Control of Noncommunicable Diseases (NCDs) 2013–2020 aimed for 80% availability of essential medicines and technologies for NCDs, with policies to improve equitable access [13]. However, significant gaps remain, especially for LMICs. For instance, only half of the eligible patients with diabetes in 55 LMICs received glucose lowering medications from the 2020 WHO Package of Essential Noncommunicable Disease Interventions (WHO PEN) [14]. Surveys conducted in 22 countries found metformin unavailable in 35% of LIC pharmacies, while insulin availability in LICs was as low as 10%, compared to 94% in HICs [15]. A meta-analysis of African studies showed overall insulin availability under 50% [16].

However, these studies did not include the recently added essential medicines, SGLT2 inhibitors and long-acting insulin [14–16]. Additionally, variations in the year of survey collection and implementation methodologies could potentially influence cross-country comparisons [14]. Studies investigating the consumption trends of newer therapies, such as SGLT2 inhibitors, DPP-4 inhibitors and GLP-1 receptor agonists, have been single-country analyses, predominantly focusing on HICs [17–22]. The inclusion of these novel drugs in clinical guidelines varies across countries, such as in 2015 in Canada [23], partially in 2014 in Singapore [24], and only in 2019 in Korea [25], highlighting the need to assess consumption trends across countries.

To address these limitations, we assessed the consumption of all drugs used in diabetes management using a single-mechanism classification framework and globally standardized database comprising sales data from 72 countries and 2 regions, collectively representing over 80% of the global population. Our analysis involved examining consumption patterns on a global scale, making comparisons across country income tiers, and ranking individual countries to identify areas of unmet need. Furthermore, we conducted a detailed examination of the specific drugs that drive the consumption of particular drug classes. The data in our study spanned a period of 12 years, from 2010 to 2021, enabling us to examine consumption trends in relation to the WHO EML additions [11] and the release of national and international treatment guidelines and consensus statements [23–34].

## Methods

### Data sources and outcomes

The consumption data for diabetes medications was obtained by utilising the IQVIA MIDAS database, a comprehensive source that has been previously used in various studies to analyse consumption patterns of antibiotics [35], opioids [36] and diabetes medications [22,37,38] by using sales data as a proxy for consumption. The IQVIA MIDAS database primarily collects data at the distribution stage, involving the transfer of medications from wholesalers, distributors, and manufacturers to dispensing facilities such as pharmacies, clinics, and hospitals. On some occasions, data collection also occurs during the distribution process to patients. To ensure reliability and validity, data collection for the MIDAS database is standardised by using established data standards, integrating core data into a centralised system with internationalised coding features and validating it against local audits. For our study, we accessed the IQVIA MIDAS quarterly sales data from the hospital and retail sectors covering the period from 2010 to 2021 across 72 countries and 2 regions. Countries included in the 2 regions were: Central America (Costa Rica, El Salvador, Guatemala, Honduras, Nicaragua, Panama) and French-speaking West Africa (Benin, Burkina Faso, Cameroon, Chad, Cote divoire, Congo, Guinea, Mali, Niger, Senegal, Togo). Each region’s data is aggregated from all its included countries.

Complete data was obtained for 70 countries and 2 regions, with the exception of Bosnia and Serbia, for which data was available from 2011 onwards (S1 Table). For the sector breakdown, data was accessible for all 72 countries and 2 regions in the retail sector, while information regarding the hospital sector was obtainable for 46 countries. However, in the case of 26 countries and 2 regions where data for the hospital sector was unavailable, it was observed that the retail sector accounted for a substantial majority, constituting at least 70% of the market share for diabetes medications in 21 countries. As a result, the potential impact of the missing hospital sector data on our consumption analysis was likely to be minimal. Moreover, as part of our sensitivity analysis, we employed a method to impute the missing hospital sector data for these 28 countries by utilizing the available retail sector data and considering the market shares of both retail and hospital sectors within each respective country (S1–S8 Figs).

In accordance with the European Pharmaceutical Marketing Research Association (EphMRA) classification, we adopted the categorization provided under A10 Drugs used in diabetes [39], including human insulins and analogues, sulphonylureas, biguanides, glitazones, alpha-glucosidase inhibitors, glinides, dipeptidyl peptidase IV (DPP-4) inhibitors, sodium-glucose transport protein 2 (SGLT2) inhibitors and glucagon like peptide 1 (GLP-1) receptor agonists. We excluded A10D animal insulins, A10E insulin devices and A10X other drugs used in diabetes. We excluded ATC codes that contain dual or multiple pharmacological profile as single-mechanism therapeutic class definitions were applied in this study. Sales data may not reflect actual consumption due to factors such as non-adherence and stockpiling. However, our study covers a long period of time (2010–2021), therefore stockpiling is unlikely to be a major issue. We used the defined daily dose (DDD) metric, which refers to the “estimated average maintenance dose per day for a drug used for its main indication in adults”, as recommended by the World Health Organization (WHO) [40]. Furthermore, the use of DDD facilitates cross-country comparisons, independent of variations in package size and drug strength [40]. Notably, the DDD metric has been consistently employed as a consumption measure in previous studies exploring the consumption patterns of diabetes medications in both multi-country comparisons [37,38,41] and single-country investigations [19,42].

To determine the consumption rate, we used defined daily doses per thousand inhabitants per day (DDD/TID) by using the formula [(annual DDD/population)*(1,000/365)]. To account for population size differences, WHO recommends reporting drug utilization using DDD per 1,000 inhabitants per day (DDD/TID), facilitating meaningful comparisons across time periods and demographic groups [43]. Our approach aligns with several previous studies—both single-country and multi-country—which have similarly used DDD/TID calculations [44–51]. The respective country’s population was sourced from the World Bank DataBank [52]. In order to classify countries into distinct income groups, namely high-income countries (HICs), upper-middle income countries (UMICs), and low- and lower-middle income countries (LMICs), we employed the 2016 World Bank income classification [53]. For individual country rankings, we utilised a 3-year interval from 2019-2021.

### Statistical analysis

We examined trends in the median consumption rate across three income groups over the period of 2010–2021. Additionally, we provided a snapshot of country rankings based on consumption rate during 3-year intervals for 2010–2012 and 2019–2021. We conducted three sub-analyses that focused on the following areas: 1) WHO essential medicines (EML), 2) insulin and 3) new drug classes, namely DPP-4 inhibitors, GLP-1 receptor agonists and SGLT2 inhibitors.

In the first sub-analysis, we focused on the strength-specific medicines listed within the 2021 WHO EML (22nd list). The following medicines were considered: 1) metformin (500mg), 2) soluble insulin and biosimilars (40IU/ml, 100IU/ml), 3) intermediate-acting insulin and biosimilars (40IU/ml, 100IU/ml), 4) gliclazide/A10BB sulphonylureas (30mg, 60mg, 80mg), 5) long-acting insulin analogues and biosimilars (100IU/ml) and 6) empagliflozin (10mg, 25mg) including therapeutic alternatives such as canagliflozin and dapagliflozin (S2 Table) [10]. In the insulin sub-analysis, we categorized the insulin types according to the EphMRA classification, which encompassed the following: 1) A10C1 fast-acting human insulin and analogues, 2) A10C2 intermediate-acting human insulin and analogues and 3) A10C5 long-acting human insulin and analogues [39]. In the third sub-analysis, we focused on the diffusion of novel drugs using the EphMRA classification [39]: 1) DPP-4 inhibitor antidiabetics, 2) A10P SGLT2 inhibitor antidiabetics and 3) A10S GLP-1 agonist antidiabetics.

For all three sub-analyses, we analysed consumption trends across the three income groups, which is crucial for understanding how income variations influence consumption behaviours. We ranked countries based on their consumption rates overall and for selected drug classes, between 2019 and 2021. For insulin and novel drug classes, we conducted additional analyses on the proportion of each insulin type or new drug class in relation to the total insulin or drug class consumption across the three income groups. Furthermore, we examined the specific drugs driving the uptakes of insulin and new drug classes. The main tables and figures appear within the text, while additional ones, referred to as SX Table or SX Fig throughout the paper, are provided in the Supporting information.

## Results

### Overall analysis

**Fig 1:**
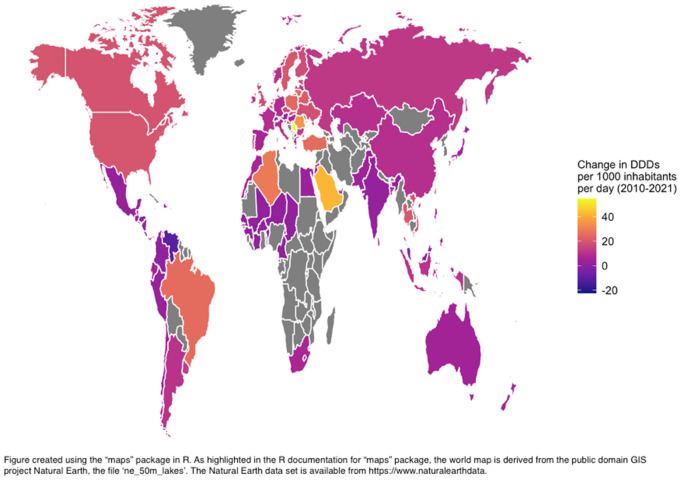
Change in consumption rate by country between 2010–2012 and 2019–2021 in DDD/TID.

From 2010-2012–2019–2021, there was a substantial increase in the global median consumption rate of glucose lowering medications, rising by 38% from 39.2 to 54.0 defined daily doses per thousand inhabitants per day (DDD/TID) (Fig 1). This notable surge can be attributed to heightened consumption in Bosnia (+284%), China (+270%), Indonesia (+249%) and Ukraine (+210%).

**Fig 2:**
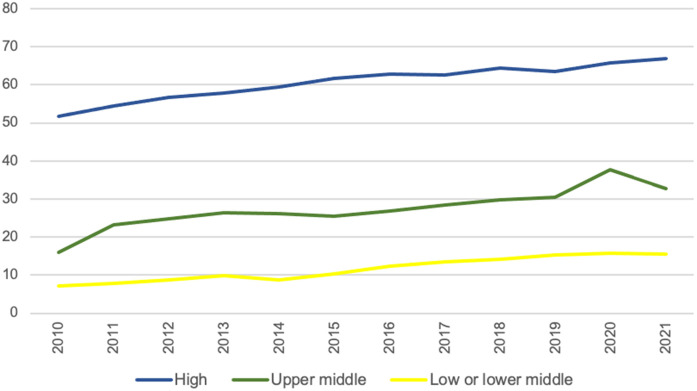
Median consumption rate by country income classification in DDD/TID (2010–2021).

Over the span of 2010–2021, the median consumption rate of glucose lowering medications in HICs was recorded at 60.1 DDD/TID [interquartile range (IQR), 46.5-70.6], in contrast to 26.9 DDD/TID [IQR, 8.0-51.3] in UMICs and 10.8 DDD/TID [IQR, 6.5-18.5] in LMICs (Fig 2). Out of global consumption of glucose lowering medications from 2019 to 2021 in terms of total DDD, HICs accounted for approximately half, while UMICs consumed roughly one-third, signifying a 9% rise from the 2010–2012 period. LMICs had the lowest consumption at 16% (S9 Fig).

**Fig 3:**
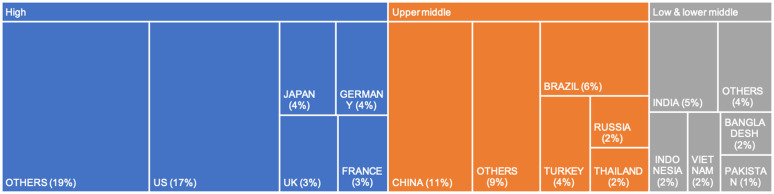
Proportion of each country consumption out of global consumption in DDD (2019–2021).

In both the 2010–2012 and 2019–2021 periods, the top five HICs collectively represented about one-third of global glucose lowering medication consumption, with the USA at 17%, Japan at 4%, Germany at 4%, UK at 3%, and France at 3% in 2019–2021 (Fig 3). Among UMICs, China’s consumption rose from 4% of global consumption in the 2010–2012 period to 11% in the 2019–2021 period (S10 Fig).

**Fig 4:**
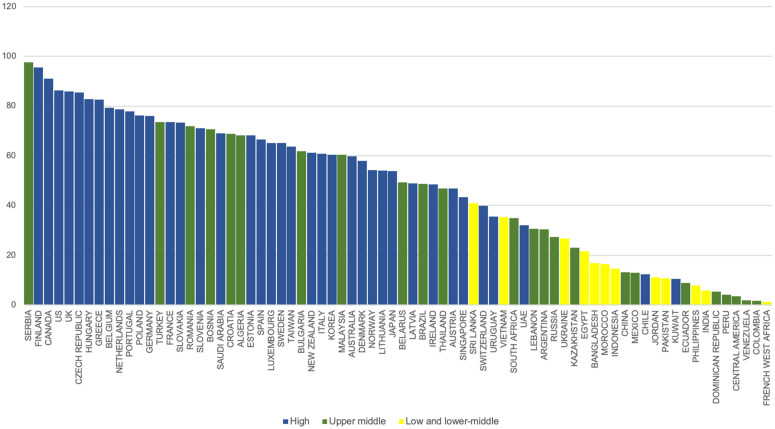
Consumption rate by country in DDD/TID (2019–2021).

During the 2010–2012 period, HICs such as Finland (78.7 DDD/TID), Hungary (75.8 DDD/TID) and Greece (75.1 DDD/TID) displayed the highest consumption rates (S11 Fig). In the 2019–2021 timeframe, Finland (95.6 DDD/TID) maintained within the top three alongside an UMIC, Serbia (97.5 DDD/TID) and a HIC, Canada (91.1 DDD/TID) (Fig 4). The lowest consumption among HICs was seen in Kuwait (10.5 DDD/TID). Consumption rates of UMICs also exhibited considerable variation across different countries: Serbia (97.5 DDD/TID), South Africa (35.0 DDD/TID) and Colombia (1.8 DDD/TID). LMIC consumption rates were lower compared to HICs and UMICs both at the beginning and end of the study period, with most countries below 30.0 DDD/TID in the 2019–2021 period.

### Essential medicines

**Fig 5:**
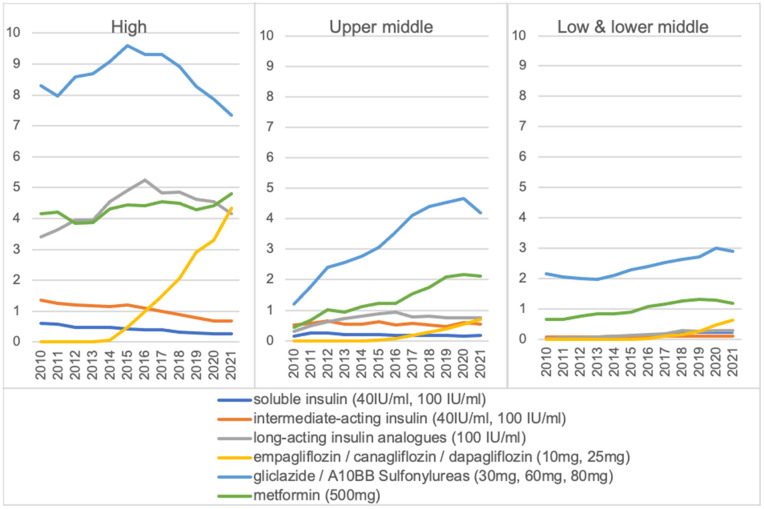
Median consumption rate of WHO EM across income groups in DDD/TID (2010–2021).

From 2010 to 2021, there was a global increase in the median consumption rate of WHO essential medicines for diabetes, except for intermediate-acting insulin (40 IU/ml, 100 IU/ml) and soluble insulin/biosimilars (40 IU/ml, 100 IU/ml) (S12 Fig). For the long-standing essential medicine metformin, although its median consumption rate rose across all three income groups, discrepancies in median consumption rate levels still exist. In the 2010–2021 period, metformin median consumption rate in HICs was 4.4 DDD/TID [interquartile range (IQR), 2.6-8.8], in contrast to 1.2 DDD/TID [IQR, 0.4-3.9] in UMICs and 0.9 DDD/TID [IQR, 0.7-1.5] in LMICs (Fig 5). As for gliclazide/A10BB sulphonylureas, which were added to the WHO Model List of Essential Medicines in 2013, the increase in median consumption rate before and after 2013 was the most pronounced in UMICs where it increased from 2.6 DDD/TID in 2013 to 4.7 DDD/TID in 2020 before decreasing in 2021. Long-acting insulin analogues and biosimilars and SGLT2 inhibitors were recently added to the WHO Model List of Essential Medicines in 2021. However, the consumption of these essential medicines exhibited an increasing trend in HICs even prior to 2021. The highest consumption rate varied substantially among income groups: long-acting insulin analogues/biosimilars (HIC: 15.0 DDD/TID in Finland; UMIC: 7.2 DDD/TID in Romania; LMIC: 0.6 DDD/TID in Ukraine) and SGLT2 inhibitors (HIC: 10.8 DDD/TID in Finland; UMIC: 6.1 DDD/TID in Turkey; LMIC: 0.8 DDD/TID in Sri Lanka). During the 2019–2021 period, Canada (56.8 DDD/TID), Malaysia (56.4 DDD/TID) and the UK (54.7 DDD/TID), displayed the highest consumption rates for all essential medicines (S13–S18 Figs).

### Insulin

**Fig 6:**
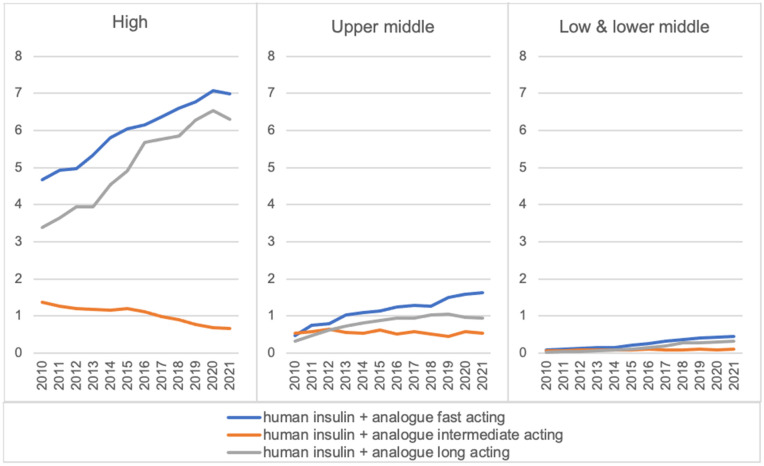
Median consumption rate by insulin type across income groups in DDD/TID (2010–2021).

From 2010 to 2021, the global median consumption rate of fast-acting and long-acting insulin increased from 1.5 to 2.3 DDD/TID and from 1.1 to 2.1 DDD/TID respectively. On the other hand, the median consumption rate of intermediate-acting insulin declined from 0.7 to 0.6 DDD/TID, driven by the reduced consumption in HICs (S12 Fig). While the median consumption rate of intermediate-acting insulin was between 0.1-1.4 DDD/TID across all income groups between 2010 and 2021, large variations were observed between HICs and other income groups for fast-acting insulin and long-acting insulin (Fig 6, S3 Table).

From 2010 to 2021, the proportion of intermediate-acting insulin out of the total insulin consumption decreased across all income groups (HICs: -8%, UMICs: -27%, LMICs: -11%) (S19 Fig). Simultaneously, the proportion of long-acting insulin increased (HICs: +10%, UMICs: +17%, LMICs: +12%). For long-acting insulin in 2021, a higher proportion of insulin detemir, at 13–14%, was utilised in HICs and UMICs, compared to LMICs at 7% (S20 Fig). Similarly, insulin degludec had broader usage in HICs, constituting 18% of total long-acting insulin consumption, while its utilisation in the other two income groups ranged from 6% to 9%. From 2010 to 2021, the proportion of fast-acting insulin out of the total insulin consumption remained roughly the same in HICs and LMICs, however, UMICs saw an increase of 11% (S19 Fig). In 2021, the usage of insulin analogues was more prevalent than insulin human base in HICs, accounting for 87% of the total fast-acting insulin consumption in 2021. UMICs followed suit with 72% usage, while LMICs exhibited a lower utilisation rate at 30% (S20 Fig).

In the 2019–2021 period, HICs, such as Finland (31.1 DDD/TID), Germany (27.9 DDD/TID) and the US (25.3 DDD/TID), displayed the highest consumption rates for all types of insulin (S21 Fig). The highest consumption rates for UMICs were seen in Romania (15.7 DDD/TID), Algeria (15.5 DDD/TID) and Turkey (15.4 DDD/TID) while for LMICs, Ukraine (4.2 DDD/TID), Egypt (1.6 DDD/TID) and Bangladesh (1.6 DDD/TID) dominated. HICs drove the consumption of fast-acting and long-acting insulin, while UMICs and LMICs drove the consumption of intermediate-acting insulin (S22–S24 Figs).

### New drug classes

**Fig 7:**
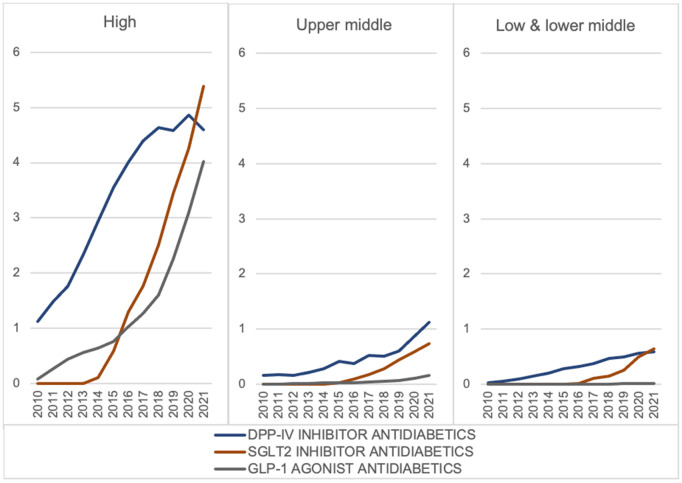
Median consumption rate of new drug classes across income groups in DDD/TID (2010–2021).

From 2010 to 2021, the median consumption rate of new drug classes rose globally, surpassing that of alpha-glucosidase inhibitors, glitazones, and glinides by 2021 (S12 Fig). However, large variations in median consumption rates of new drug classes were observed between HICs and other income groups (Fig 7, S3 Table). In 2021, the median consumption rate of SGLT2 inhibitors surpassed that of DPP-4 inhibitors, becoming the leading new drug class in HICs and LMICs, but not in UMICs.

The proportion of new drug classes out of all drug classes increased across all income groups from 2010 to 2021 (HICs: +21%; UMICs: +12%; LMICs: +11%; S25 Fig). This increase was primarily driven by all three new drug classes in HICs, whereas for UMICs and LMICs, it was driven by DPP-4 inhibitors and SGLT2 inhibitors. The specific drugs driving the change varied among income groups. For instance, for DPP-4 inhibitors in 2021, a higher proportion of sitagliptin (50%) was utilised in HICs, compared to 35% in UMICs and 27% in LMICs (S26 Fig). For SGLT2 inhibitors, empagliflozin drove the growth in HICs, whereas dapagliflozin played a more prominent role in UMICs and LMICs. Dulaglutide drove the growth of GLP-1 receptor agonist consumption in all income groups (HICs: 51%; UMICs: 31%; LMICs: 43%), however, semaglutide usage was higher in HICs and UMICs (HICs: 31%; UMICs: 29%; LMICs: 12%) while liraglutide usage was higher in LMICs (HICs: 15%; UMICs: 32%; LMICs: 45%).

During the 2019–2021 period, HICs remained as the dominant consumers of new drug classes; DPP-4 inhibitor consumption was dominated by Asian HICs while SGLT2 inhibitor and GLP-1 receptor agonist consumption was dominated by European and North American HICs (S27–S29 Figs). Overall, consumption rates were the highest in Finland (29.6 DDD/TID), Luxembourg (26.4 DDD/TID) and Japan (25.9 DDD/TID). In contrast, the highest consumption rate among UMICs was considerably lower at 13.6 DDD/TID in Turkey, and among LMICs at 5.4 DDD/TID in Sri Lanka (S30 Fig). From 2010 to 2021, Japan reported a substantial increase in DPP-4 inhibitor consumption during the first half of the 12-year timeframe whereas Singapore’s surge in usage was in the second half (S31 Fig). For SGLT2 inhibitors, Luxembourg lost its leading position in 2019, subsequently outpaced by other countries such as Canada, Finland, Portugal and Singapore (S31 Fig).

## Discussion

Our analysis revealed a substantial increase in the global consumption of glucose lowering medications from 2010 to 2021, reflecting shifts in diabetes management due to rising prevalence and expanded treatments. This trend has major policy implications, particularly for equitable access, resource allocation, and healthcare sustainability. Ensuring affordable medication access in LMICs is crucial as their diabetes burden grows. Additionally, prioritizing diabetes prevention, early intervention, and patient education can reduce future treatment demands and financial strain. A notable spike in median consumption rates was observed in UMICs in 2020, primarily in countries like Lebanon, South Africa, Brazil, Belarus, and Turkey. This surge may have been influenced by COVID-19-related stockpiling, particularly in Lebanon, where the financial crisis compounded the issue [54]. However, in Brazil, Belarus, and Turkey, the increase continued into 2021, suggesting that 2020 was not merely an anomaly but part of a sustained trend.

HICs consistently displayed the highest consumption rates throughout the study period, with UMICs and LMICs showing comparatively lower rates. This contrasted with the higher diabetes burden, in terms of prevalence, DALY and deaths, seen in UMICs and LMICs compared to HICs (S4 and S5 Tables). These disparities highlight significant inequities in healthcare access between country income groups. In many LMICs, access to glucose lowering medications is limited by economic constraints, inadequate healthcare infrastructure and fragmented supply chains, which collectively limit availability and affordability [55,56]. They may also lack the resources to implement and enforce clinical guidelines and robust public health policies, leading to suboptimal treatment practices and reduced medication accessibility [55]. Successful policy interventions have demonstrated that improving medication accessibility and clinical outcomes is possible through targeted subsidies, price negotiations with pharmaceutical companies and biosimilar policies. Brazil’s large-scale subsidising program of prescription drugs led to a reduction in hospitalisation rates by diabetes [57]. Conversely, an increase in patient co-payment level for T2D non-insulin medications in Finland led to poorer glycaemic control [58]. Pricing strategies such as value-based pricing, volume-based pricing as well as biosimilar policies enhance affordability. For instance, U.S. biosimilar insulin approvals lowered prices [59] and expanding biosimilar production in UMICs and LMICs have reduced insulin analogue costs [38].

In our analysis, the countries with the most substantial changes in consumption of glucose lowering medications between the periods 2010–2012 and 2019–2021 were UMICs and LMICs such as Bosnia, China, Indonesia and Ukraine. These marked changes can be attributed to the significant diabetes burden that these countries face, as seen in S5 Table. For instance, Bosnia and Indonesia ranked among the top 10th and 40th countries, out of 204 assessed, in terms of changes in death rates due to diabetes between 1990 and 2021 [60]. On the other hand, the highest consumption rates during the 2019–2021 period were driven by HICs such as Finland, Canada, USA, UK and UMICs such as Serbia. Canada experienced a 184% increase in diabetes prevalence from 1990 to 2021, ranking 5th among 204 countries [60] while the UK ranked 15th (154% increase) and the USA ranked 19th (141% increase) [60]. Finland and Serbia also saw notable prevalence increases (90% and 62% respectively) [60]. In the case of Serbia, which, despite being an UMIC, has effectively implemented a comprehensive diabetes management program to address the escalating diabetes burden. This program features a national registry, screening initiatives, healthcare provider education, and patient self-management strategies, likely contributing to its high medication uptake and improved diabetes care [61,62].

In our sub-analyses, the median consumption rate of WHO essential medicines for diabetes increased except for intermediate-acting insulin and soluble insulin/biosimilars. Consumption of sulphonylureas increased rapidly in UMICs after its addition to the WHO EML in 2013, while the consumption of long-acting insulin analogues increased in HICs even before their inclusion. HICs drove the consumption of fast-acting and long-acting insulin, whereas UMICs and LMICs drove the consumption of intermediate-acting insulin. For new drug classes, HICs were the dominant consumers; DPP-4 inhibitor consumption was led by Asian HICs, while SGLT2 inhibitor and GLP-1 receptor agonist consumption was dominated by European and North American HICs.

Despite the WHO EML’s long-standing role in improving access to medicines, disparities persist, even for low-cost medications such as metformin and sulphonylureas as highlighted in our study [63]. The Prospective Urban Rural Epidemiology (PURE) study, involving participants from 110,803 households and pharmacies in 22 countries, revealed affordability and availability challenges: 0.7% of HIC households faced difficulties affording metformin, in contrast to 26.9% of LIC households, where availability ranged from 65% in LICs to 100% in HICs [15]. Moreover, a meta-analysis of 21 studies conducted in Africa found metformin availability under 50% [16], reiterating the challenges in accessing essential medications in certain regions. Improving access to essential medicines in LMICs could significantly reduce the burden of diabetes, such as by prioritising additional funding for diabetes care, optimising supply chains and introducing nationwide management programs. Continuous monitoring and research are crucial to inform public health strategies, ensuring that interventions remain effective and equitable. Acknowledging cost concerns, consensus statement for South Asia and WHO 2018 clinical guidelines for low-resource settings recommend sulphonylureas due to affordability [26,27]. A review of 33 national and international guidelines found that sulphonylureas were recommended in UMICs such as Colombia in 2016, China in 2019, and Romania in 2020 [28]. These recommendations align with the observed higher utilisation of sulphonylureas in UMICs in our study.

For newer medications such as SGLT2 inhibitors and long-acting insulin analogues, added to the WHO EML in 2021, some regional and national guidelines adopted them earlier than WHO guidelines. For instance, the 2019 consensus report published by the American Diabetes Association (ADA) and the European Association for the Study of Diabetes (EASD) recommends SGLT2 inhibitors for treating high-risk patients to reduce major adverse cardiovascular events (MACE), hospitalization for heart failure (hHF), cardiovascular death, or chronic kidney disease (CKD) progression, irrespective of their HbA1c levels [29]. Similarly, Canada incorporated SGLT2 inhibitors in a 2015 interim update after approving canagliflozin and dapagliflozin [23]. This might explain the increasing trend of SGLT2 inhibitor consumption in HICs observed in our study before their inclusion in the WHO EML. These examples emphasise the importance of regional and national guidelines in swiftly incorporating new drug classes into diabetes management protocols, given their potential impact on improving patient outcomes.

Long-acting insulin analogues have become a preferred diabetes treatment due to hypoglycaemia risk, greater convenience and improved quality of life, as compared to human NPH insulin [11]. As seen in a 2020 Cochrane review, insulin glargine and insulin detemir led to fewer hypoglycaemia events than NPH insulin [64]. As a testament to their clinical value, long-acting insulin analogues have been included in the WHO’s 2021 Model List of Essential Medicines [11]. Consequently, their consumption has increased while the consumption of intermediate-acting insulin has declined, as demonstrated in our analysis. This trend is consistent with studies from Canada [65], Ireland [66] and the United States [67], all showing rising long-acting insulin use alongside declines in other insulin types like human insulin or premixed insulin. Nevertheless, this observed trend is not mirrored in UMICs and LMICs due to the substantial cost difference between insulin analogues and human insulin. While the WHO states human insulin is equally effective as analogues, analogues are at least 1.5 times more expensive, sometimes reaching threefold [68]. Our study highlights variations in analogue versus human insulin consumption across income groups, mainly due to affordability challenges. A 2016 analysis of 13 LMICs revealed that the availability of human insulin was between 55–80% while that of analogue insulin was between 55–63%. Median government prices for human insulins were $5, while for long-acting analogues, they were substantially higher at $33. A national-scale survey in Pakistan found that insulin analogues were 72.8% more expensive than human insulin [69] while in a leading hospital in Kenya, insulin glargine was priced 3.4 times higher than standard human insulin [70]. Biosimilars offer a promising solution, with WHO’s prequalification program driving competition and reducing prices. In Bangladesh, India, and Malaysia, local biosimilar production has improved affordability, expanding access to insulin analogues [38].

Recent trials have demonstrated favourable cardiovascular outcomes for GLP-1 receptor agonists [71–75], while SGLT2 inhibitors [76–79] benefit both cardiovascular and renal health, reducing the incidence of MACE, including myocardial infarction (MI), heart failure-related hospitalisation and cardiovascular mortality [80]. As a result, T2DM clinical guidelines in multiple countries have been updated to reflect the clinical benefits of these new drug classes, such as in the US, the UK and Australia [30–32]. Despite higher drug costs, there is an increasing use of novel agents. This trend highlights the growing emphasis on comprehensive diabetes care, which involves managing cardiovascular and renal risk factors beyond glycaemic control, and the associated cost reductions due to lowered diabetes complications. However, this positive trend is predominantly observed in HICs. Among the Nordic countries, Finland and Denmark lead in new drug class consumption but not Sweden. This distinction could be due to the differences in their treatment guidelines. While the treatment guidelines in Finland and Denmark include newer medications such as DPP-4 inhibitors and GLP-1 receptor agonists (with Denmark also including SGLT-2 inhibitors), Sweden recommends sulphonylureas, insulin, or repaglinide in the second line [33]. Canada, another frontrunner, also updated its treatment guidelines in 2013 and 2015, incorporating new drug classes to reflect their clinical benefits [23]. The notable increase of new drug classes in Asian countries seen in our analysis is corroborated by several other studies. A study using Japan’s National Health Insurance data (2014–2017) found DPP-4 inhibitors were the most prescribed first-line diabetes drug (65.1%), followed by biguanides (15.9%) and SGLT2 inhibitors [20]. Japan’s 2016 guidelines offered flexible treatment options based on patient characteristics [34]. Similarly, Taiwan’s NHIRD data (2008–2013) showed a substantial rise in DPP-4 inhibitor use [19]. However, in UMICs and LMICs, access remains limited due to cost and availability. Further research is needed to understand adoption barriers in these regions.

Our study showed that the consumption of various new drug classes is dominated by different regions. This can be attributed to two key factors. Firstly, DPP-4 inhibitors may be more effective in Asians, as increased DPP4 enzyme activity has been observed in Asian Indian T2DM patients [81]. Furthermore, a study reported greater reductions in HbA1c levels with linagliptin, a DPP-4 inhibitor, in Asians compared to Caucasians [81]. Secondly, treatment guidelines in Asia may not be updated as swiftly. For instance, Singapore’s Ministry of Health (MOH) clinical practice guidelines recommended DPP-4 inhibitors in 2014 but have not yet been updated to include the other two drug classes [24]. Similarly, Korea’s Clinical Practice Guidelines for T2DM was updated only in 2019 to incorporate evidence on SGLT-2 inhibitors and GLP-1 receptor agonists [25]. In contrast, Canadian guidelines were updated by 2015 to include all three new drug classes. These regional disparities are critical, as emerging evidence suggests that SGLT-2 inhibitors and GLP-1 receptor agonists show superiority over DPP-4 inhibitors in reducing the risk of most cardiorenal outcomes. They are increasingly regarded as preferred treatments for T2DM and cardiorenal diseases, as supported by a network meta-analysis of 23 cardiovascular and renal outcome trials [82]. These differences in the adoption of new drug classes highlight the need for continuously updated treatment guidelines to optimise diabetes management and improve patient outcomes globally.

It is worth taking into account limitations of the present study when interpreting our analysis. Firstly, individual country data was not available for Central America and French-speaking West Africa. However, our analysis covered more than 80% of the global population. Secondly, we used the data from national-level sales database, therefore, we could not distinguish within-country variations, prescribing patterns, or usage in specific lines of treatment. We also assumed drugs sales to be a proxy for consumption. Additionally, real-world drug dosing may differ from the standardized DDD values. Thirdly, ATC codes with dual or multiple pharmacological profile were excluded from the analysis. For example, although premixed insulins are recognised as distinct categories in the ATC system and have been widely used throughout the study period, they combine both basal and bolus components, which does not align with the single-mechanism therapeutic class categorisation applied in this study. Their exclusion should be considered when interpreting the study findings and future research could investigate utilisation patterns of these drug categories to complement the broader class-level trends described in this paper. Fourth, drug classes such as GLP-1 receptor agonists have other indications apart from diabetes, such as weight loss in obese patients [83], which could potentially influence the results. Fifth, the available data does not permit differentiation between type 1 and type 2 diabetes. Sixth, country-specific regulations may have influenced the adoption of medicines apart from affordability challenges.

In conclusion, our study is the first to present consumption trends of glucose lowering medications on a global scale, encompassing data from 74 countries and enabling cross-country comparisons of essential medicines, insulin classes, and novel drug classes. While acknowledging the study’s limitations, the findings shed valuable light on the current landscape of glucose lowering medication usage. Future research should explore within-country variations to identify local barriers and facilitators, as well as trends in biosimilar medications. Additionally, there is a need to assess the long-term impact of novel drug classes. Addressing disparities in medication access, particularly in UMICs and LMICs, is essential to improving diabetes care. Optimising global diabetes management requires regular updates to national guidelines and the WHO Essential Medicines List, incorporating emerging evidence and cost-effectiveness considerations. Policy interventions, including pricing strategies and funding priorities, will be vital in addressing affordability challenges.

## Supporting information

S1 Fig Consumption rate by country in DDD/TID (2019–2021), after imputing missing hospital sector data. (TIF)

S2 Fig Median consumption rate by country income classification in DDD/TID (2010–2021), after imputing missing hospital sector data. (TIF)

S3 Fig Median consumption rate of WHO EM across income groups in DDD/TID (2010–2021), after imputing missing hospital sector data. (TIF)

S4 Fig Median consumption rate by insulin type across income groups in DDD/TID (2010–2021), after imputing missing hospital sector data. (TIF)

S5 Fig Proportion of insulin types consumed out of total insulin consumption across income groups in DDD (2010 & 2021), after imputing missing hospital sector data. (TIF)

S6 Fig Median consumption rate of new drug classes across income groups in DDD/TID (2010–2021), after imputing missing hospital sector data, after imputing missing hospital sector data. (TIF)

S7 Fig Proportion of drug classes consumed out of total drug consumption across income groups in DDD (2010 & 2021), after imputing missing hospital sector data. (TIF)

S8 Fig Consumption rate trends of new drug classes of HICs with the top six highest consumption rates in 2019–2021 period in DDD/TID (2010–2021), after imputing missing hospital sector data. (TIF)

S9 Fig Proportion of each income group consumption out of global consumption in DDD (2010–2012, 2019–2021). (TIF)

S10 Fig Proportion of each country consumption out of global consumption in DDD (2010–2012). (TIF)

S11 Fig Consumption rate by country in DDD/TID (2010–2012). (TIF)

S12 Fig Median consumption rate by EM/insulin type/drug class for all countries in DDD/TID (2010–2021). (TIF)

S13 Fig Consumption rate for WHO EM by country in DDD/TID (2019–2021). (TIF)

S14 Fig Consumption rate for metformin (500mg) by country in DDD/TID (2019–2021). (TIF)

S15 Fig Consumption rate for long-acting insulin analogues (100 IU/ml) by country in DDD/TID (2019–2021). (TIF)

S16 Fig Consumption rate for empagliflozin/ canagliflozin/ dapagliflozin (10mg, 25mg) by country in DDD/TID (2019–2021). (TIF)

S17 Fig Consumption rate for soluble insulin (40IU/ml, 100 IU/ml) by country in DDD/TID (2019–2021). (TIF)

S18 Fig Consumption rate for gliclazide/ A10BB Sulfonylureas (30mg, 60mg, 80mg) by country in DDD/TID (2019–2021). (TIF)

S19 Fig Proportion of insulin types consumed out of total insulin consumption across income groups in DDD (2010 & 2021). (TIF)

S20 Fig Proportion of individual drugs in each insulin type across income groups (2021). (TIF)

S21 Fig Consumption rate for insulin by country in DDD/ TID (2019–2021). (TIF)

S22 Fig Consumption rate for fast-acting human insulin+analogue by country in DDD/ TID (2019–2021). (TIF)

S23 Fig Consumption rate for intermediate-acting human insulin+analogue by country in DDD/ TID (2019–2021). (TIF)

S24 Fig Consumption rate for long-acting human insulin+analogue by country in DDD/ TID (2019–2021). (TIF)

S25 Fig Proportion of drug classes consumed out of total drug consumption across income groups in DDD (2010 & 2021). (TIF)

S26 Fig Proportion of individual drugs in new drug classes across income groups (2021). (TIF)

S27 Fig Consumption rate for DPP-IV inhibitors by country in DDD/ TID (2019–2021). (TIF)

S28 Fig Consumption rate for SGLT2 inhibitors by country in DDD/TID (2019–2021). (TIF)

S29 Fig Consumption rate for GLP-1 agonists by country in DDD/TID (2019–2021). (TIF)

S30 Fig Consumption rate for new drug classes by country in DDD/ TID (2019–2021). (TIF)

S31 Fig Consumption rate trends of new drug classes of HICs with the top six highest consumption rates in 2019–2021 period in DDD/TID (2010–2021). (TIF)

S1 Table IQVIA MIDAS database data availability (2010–2021). (PDF)

S2 Table WHO Model List of Essential Medicines (2021) and WHO Essential Medicines included in the study. (PDF)

S3 Table Changes in consumption rate between 2010 and 2021 across income groups. (PDF)

S4 Table Median diabetes prevalence rate, median diabetes age-standardised DALY rate, median diabetes death rate and median consumption rate of glucose lowering medications in 2021. DDD/TID: defined daily doses per thousand inhabitants per day; DALY: disability-adjusted life year.
(PDF)

S5 Table Median percentage changes in diabetes prevalence rate (1990–2021), diabetes age-standardised DALY rate (1990–2021), diabetes death rate (1990–2021) and consumption rate of glucose lowering medications (2010–2021). DALY: disability-adjusted life year.
(PDF)
