# Supplementary figures and images for: Trends in global glucose lowering medication consumption: Insights from pharmaceutical sales data (2010–2021)
Source: PLOS Glob Public Health. 2025 Oct 22;5(10):e0005326. doi: 10.1371/journal.pgph.0005326 (PMC12543110; doi:10.1371/journal.pgph.0005326)

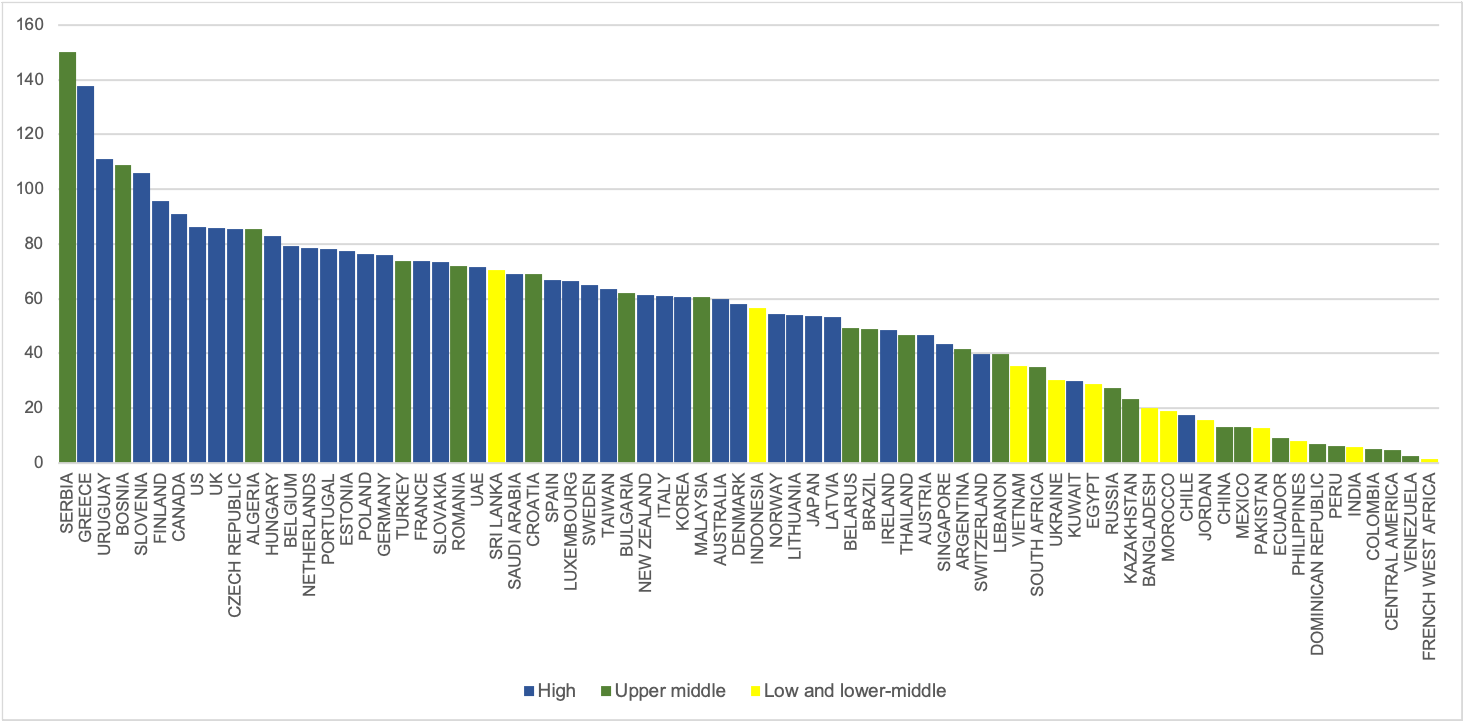

Supplement: S1 Fig — (TIF) [file pgph.0005326.s001.tif]

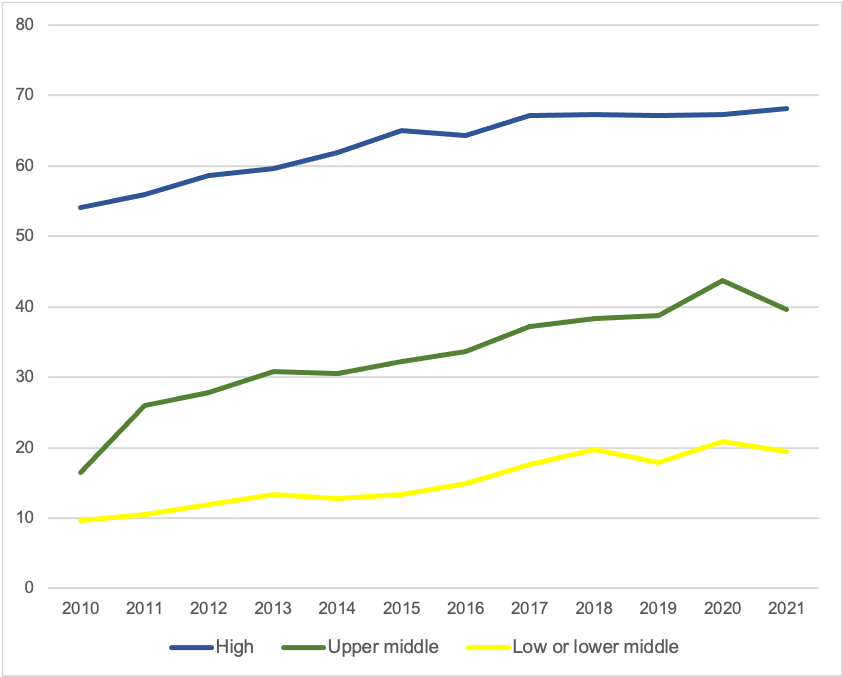

Supplement: S2 Fig — (TIF) [file pgph.0005326.s002.tif]

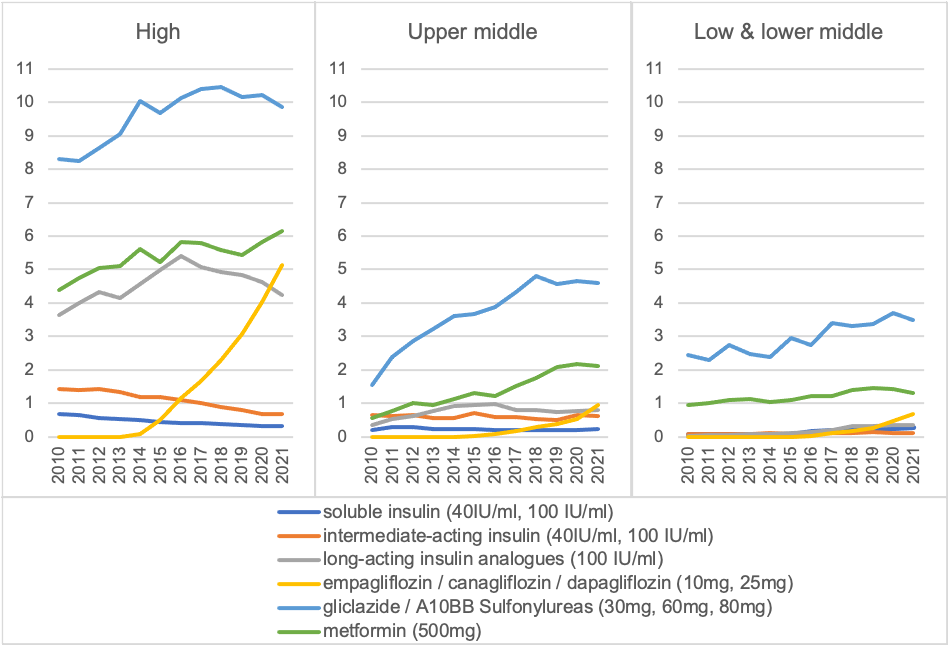

Supplement: S3 Fig — (TIF) [file pgph.0005326.s003.tif]

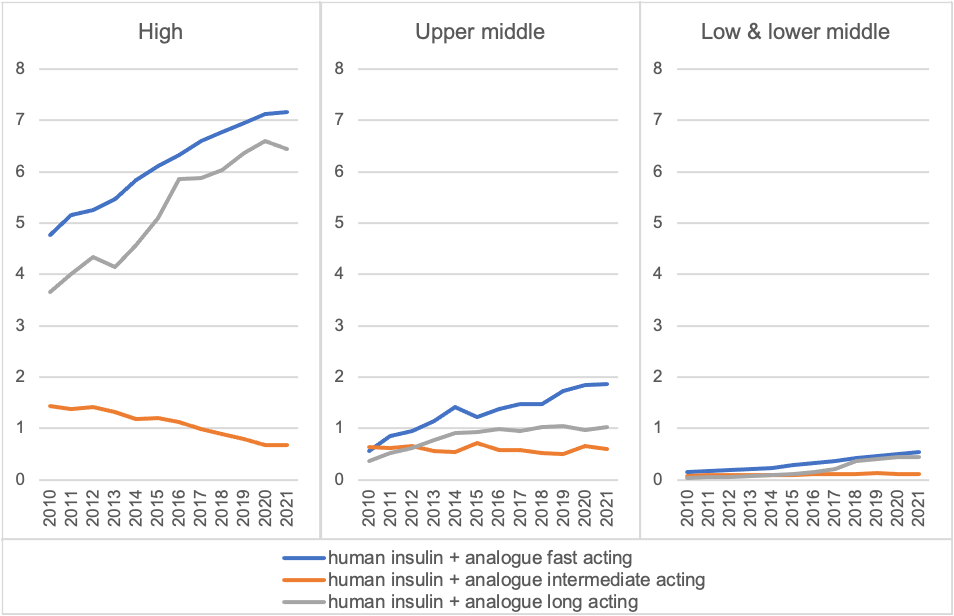

Supplement: S4 Fig — (TIF) [file pgph.0005326.s004.tif]

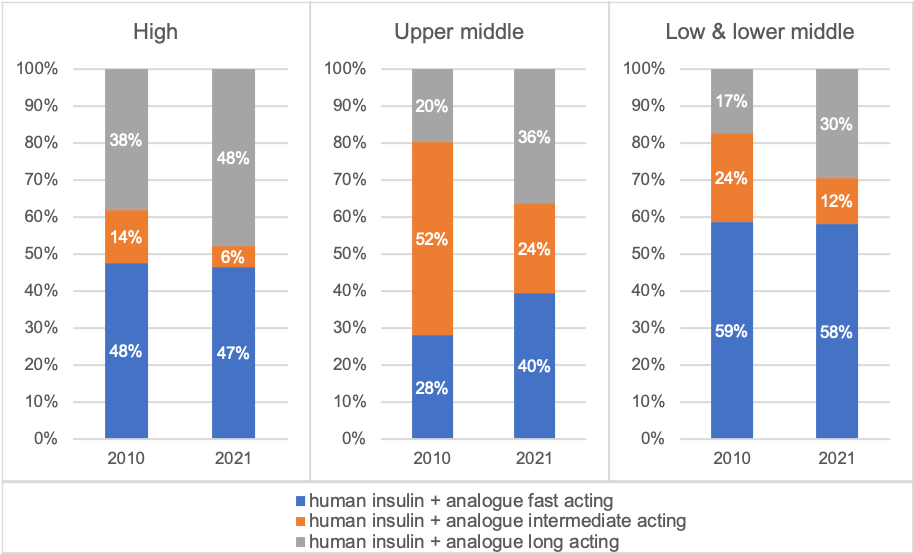

Supplement: S5 Fig — (TIF) [file pgph.0005326.s005.tif]

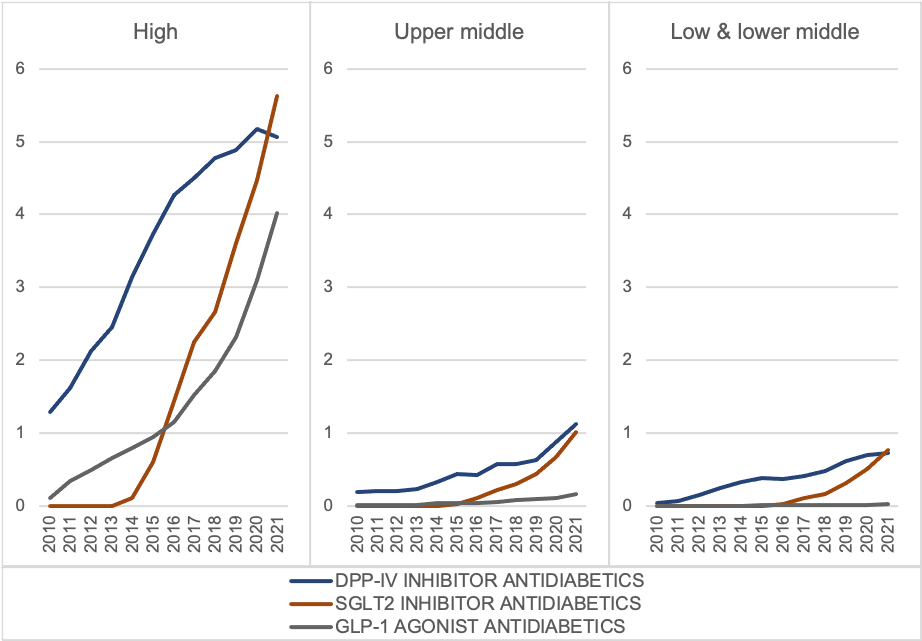

Supplement: S6 Fig — (TIF) [file pgph.0005326.s006.tif]

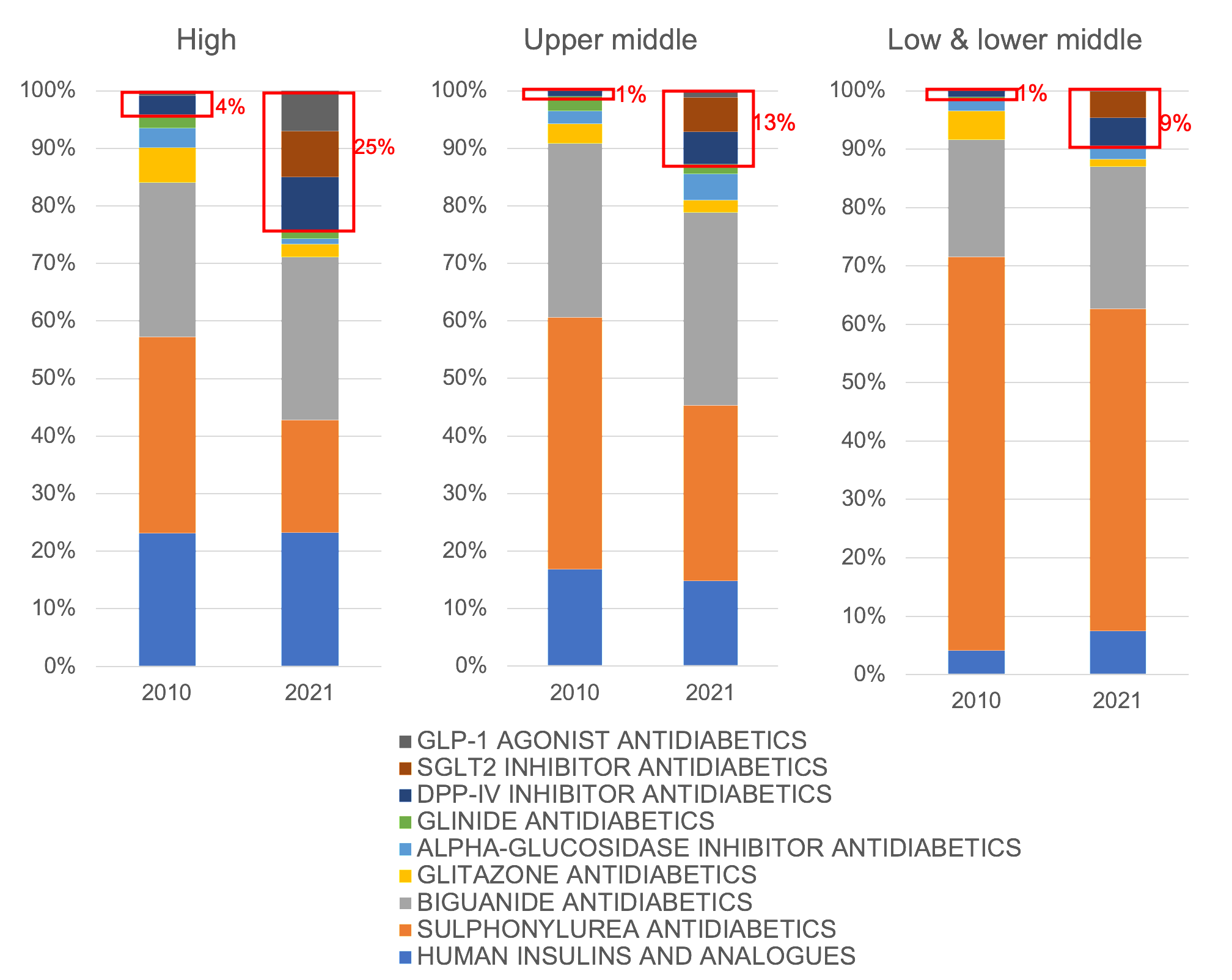

Supplement: S7 Fig — (TIF) [file pgph.0005326.s007.tif]

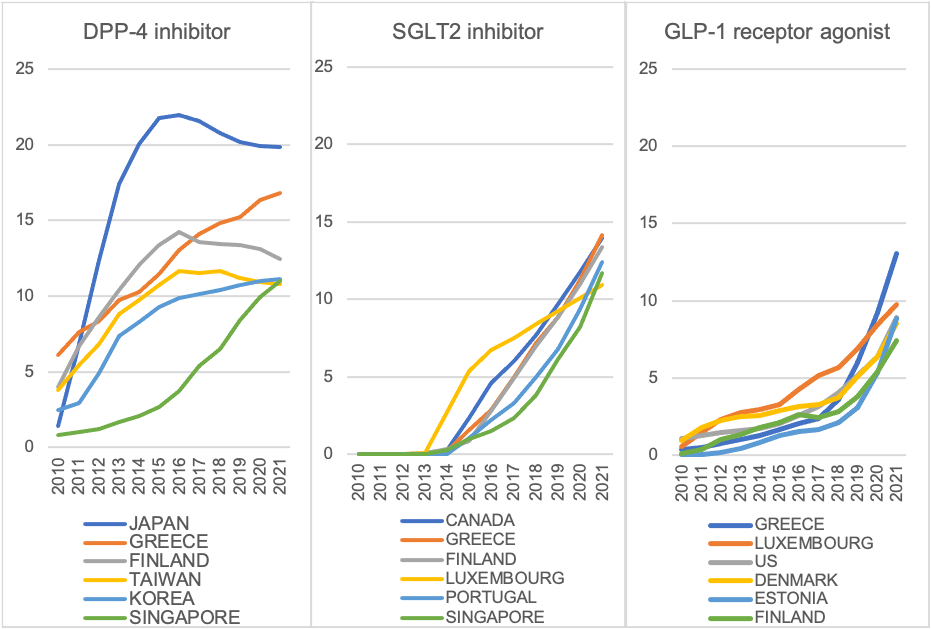

Supplement: S8 Fig — (TIF) [file pgph.0005326.s008.tif]

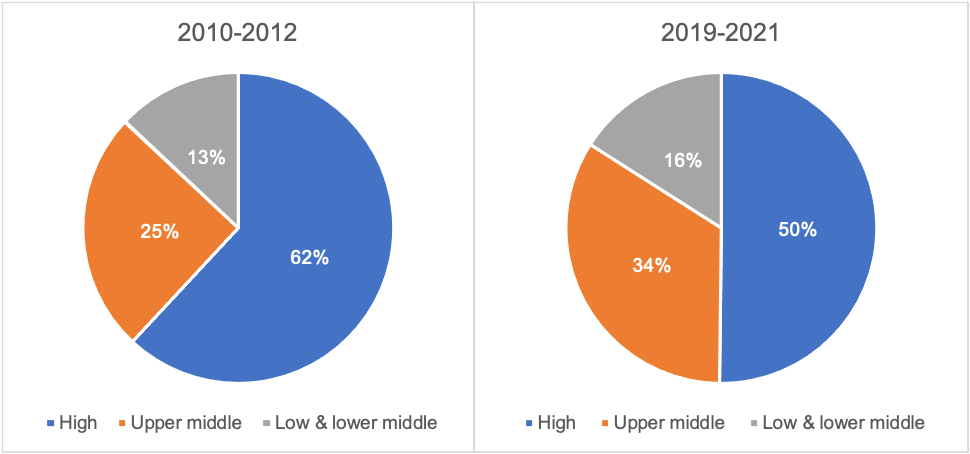

Supplement: S9 Fig — (TIF) [file pgph.0005326.s009.tif]

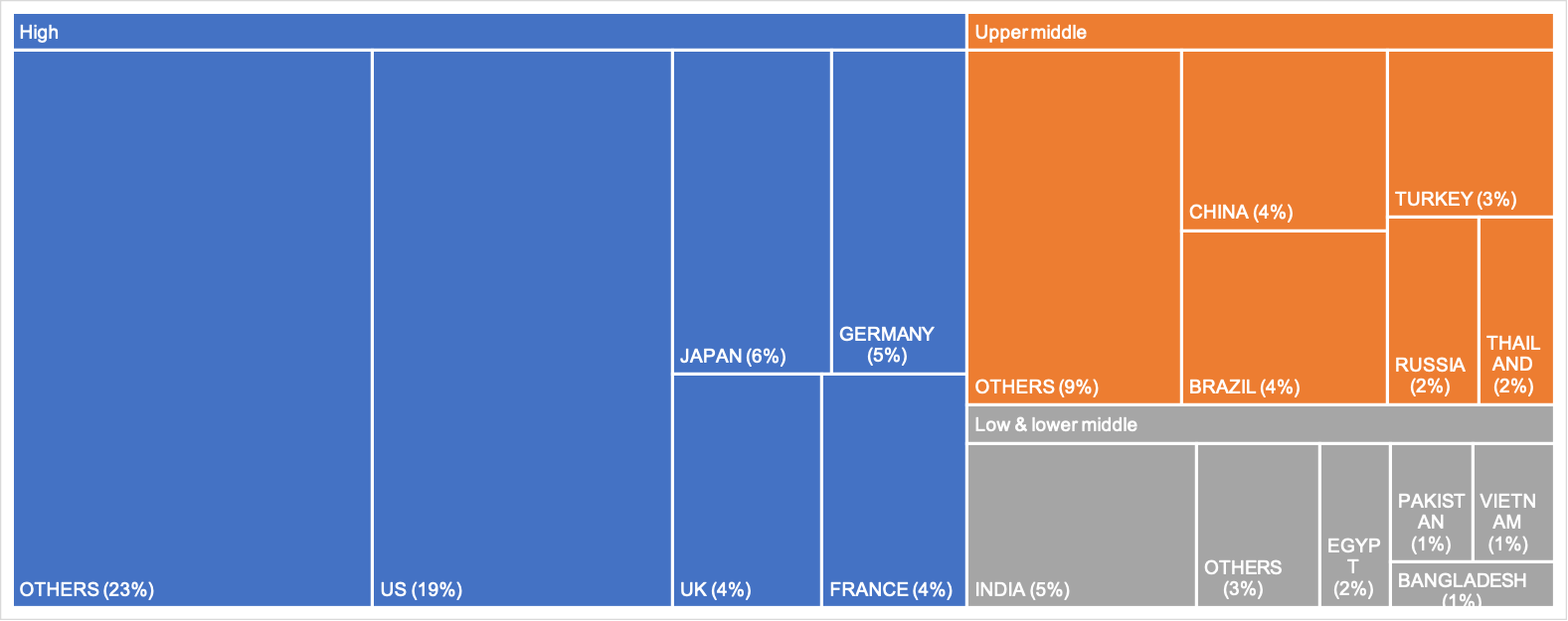

Supplement: S10 Fig — (TIF) [file pgph.0005326.s010.tif]

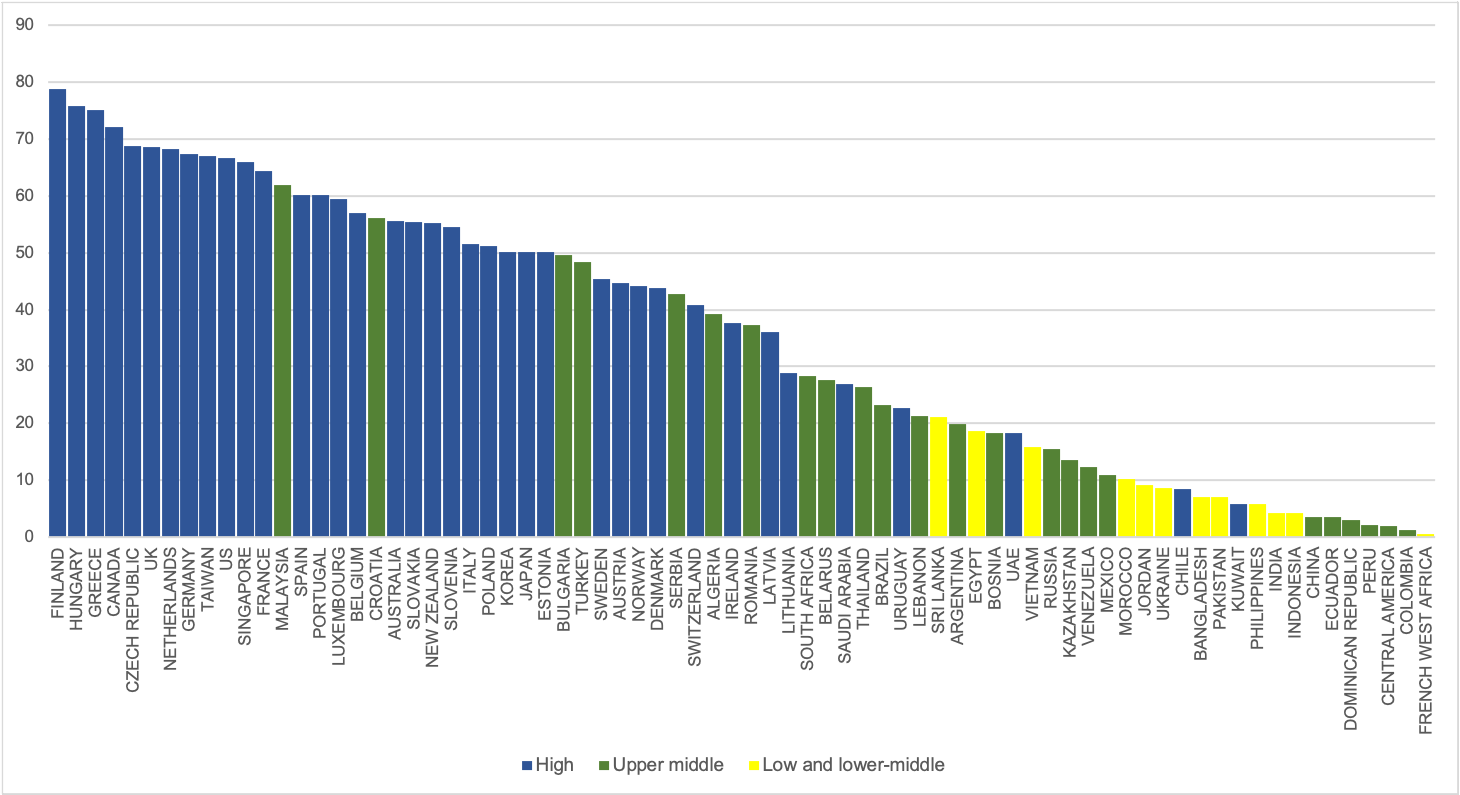

Supplement: S11 Fig — (TIF) [file pgph.0005326.s011.tif]

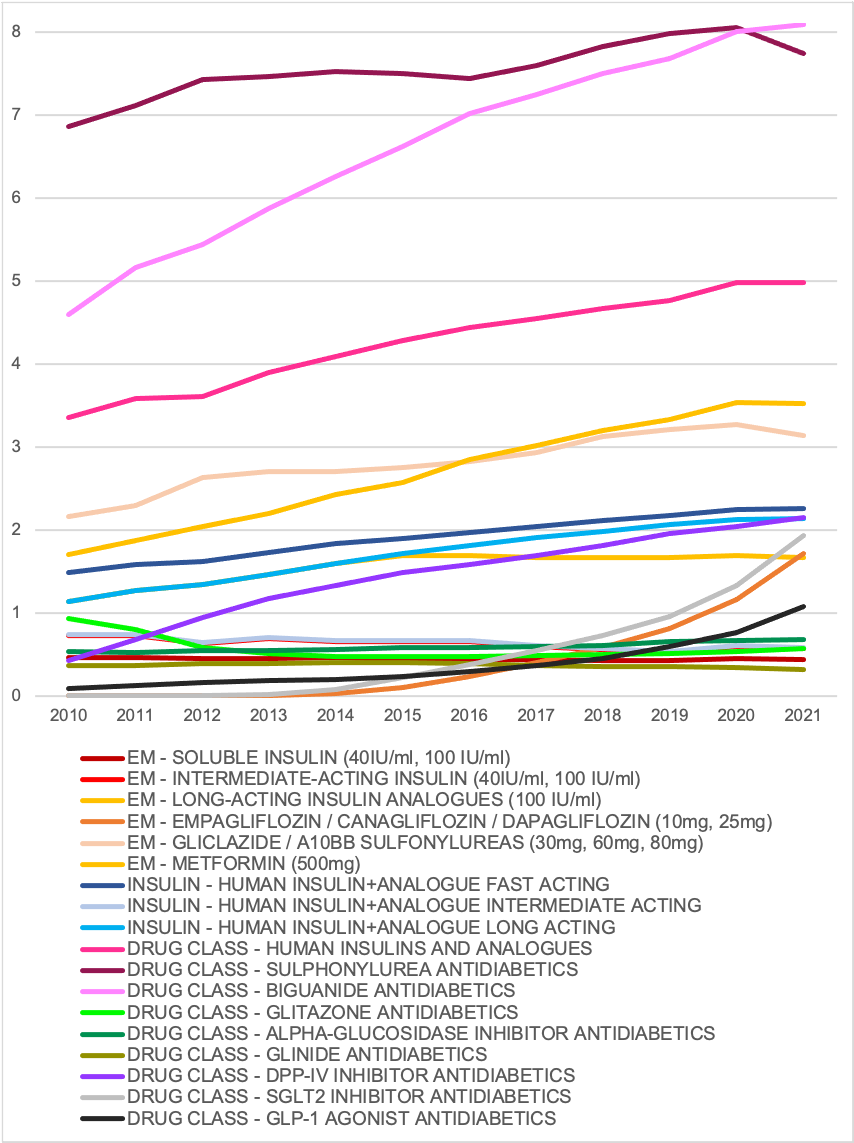

Supplement: S12 Fig — (TIF) [file pgph.0005326.s012.tif]

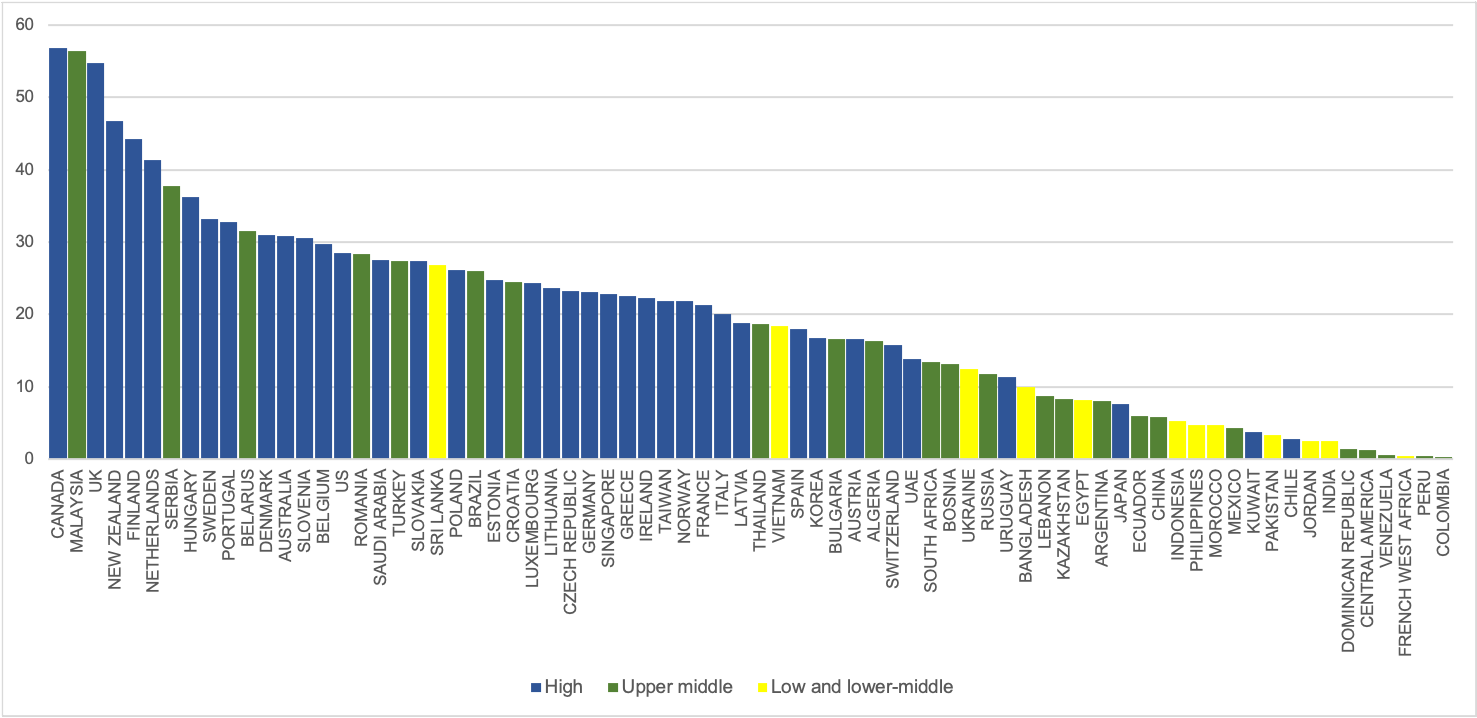

Supplement: S13 Fig — (TIF) [file pgph.0005326.s013.tif]

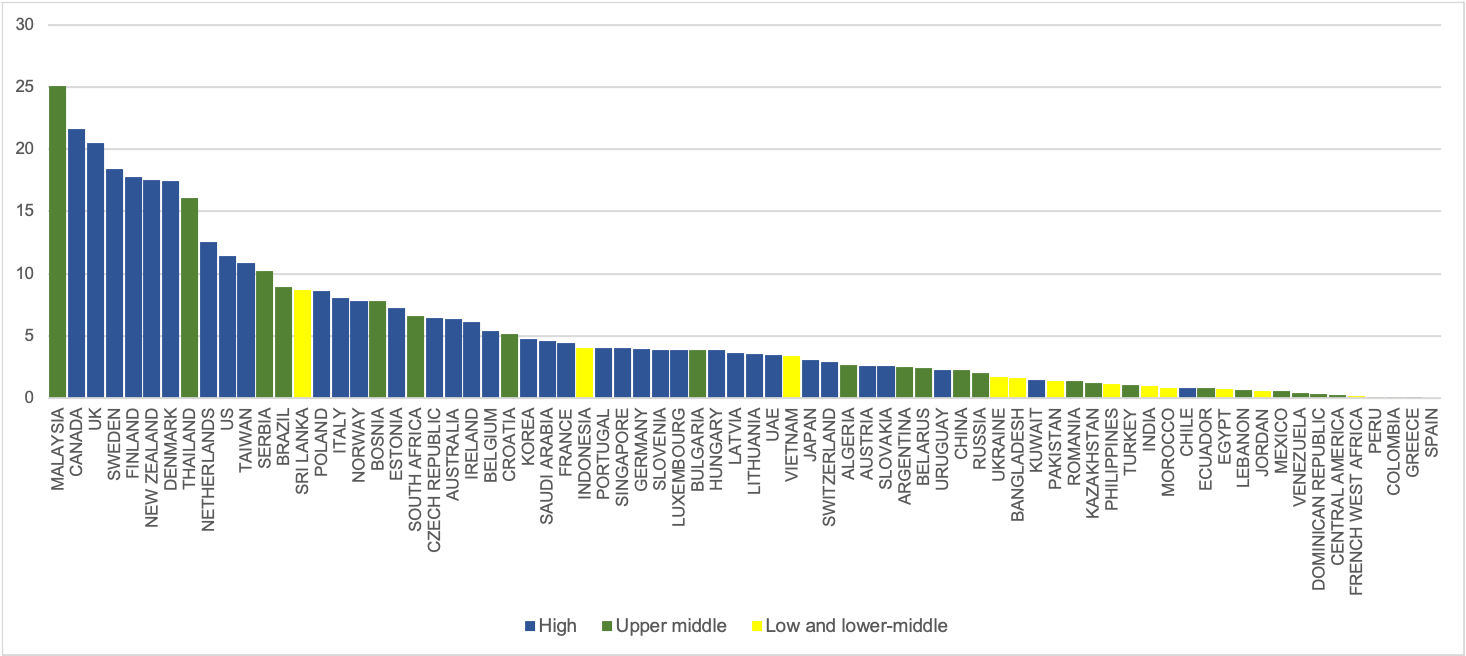

Supplement: S14 Fig — (TIF) [file pgph.0005326.s014.tif]

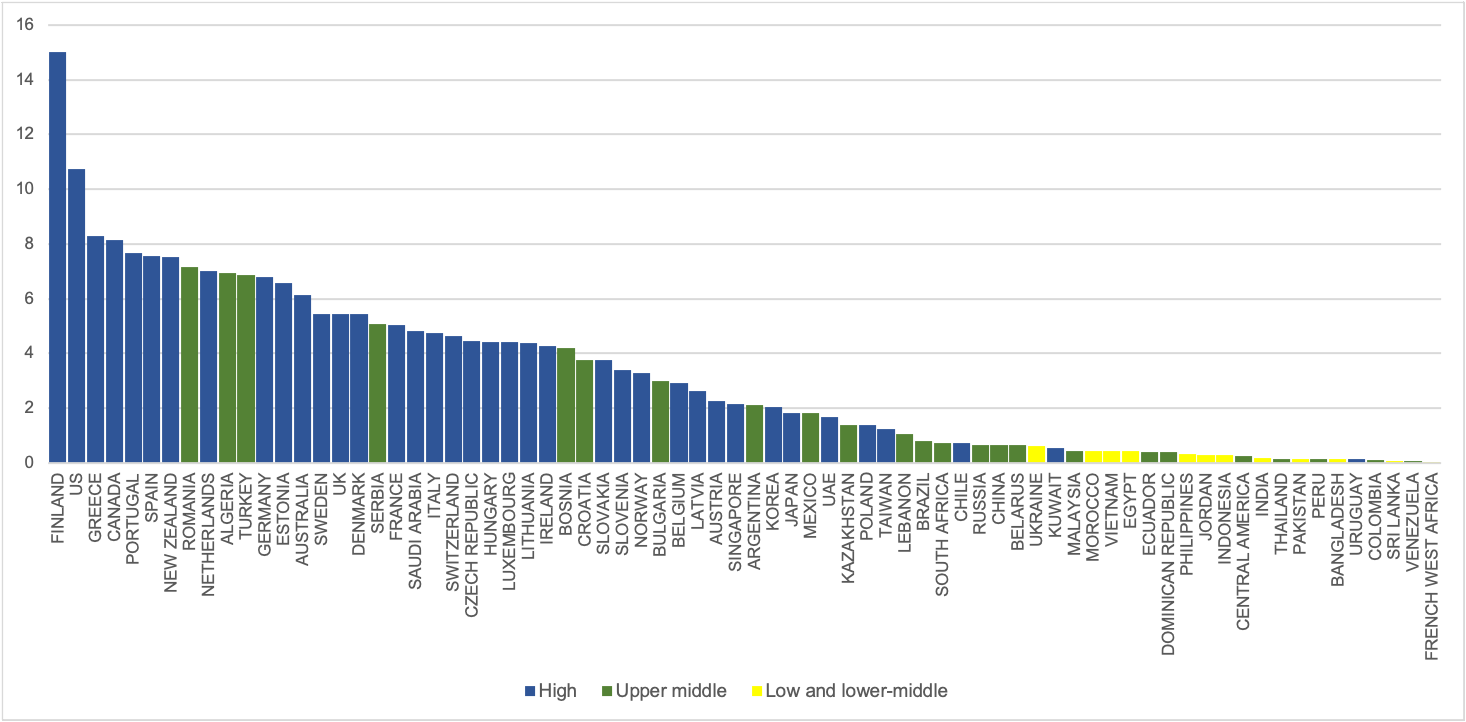

Supplement: S15 Fig — (TIF) [file pgph.0005326.s015.tif]

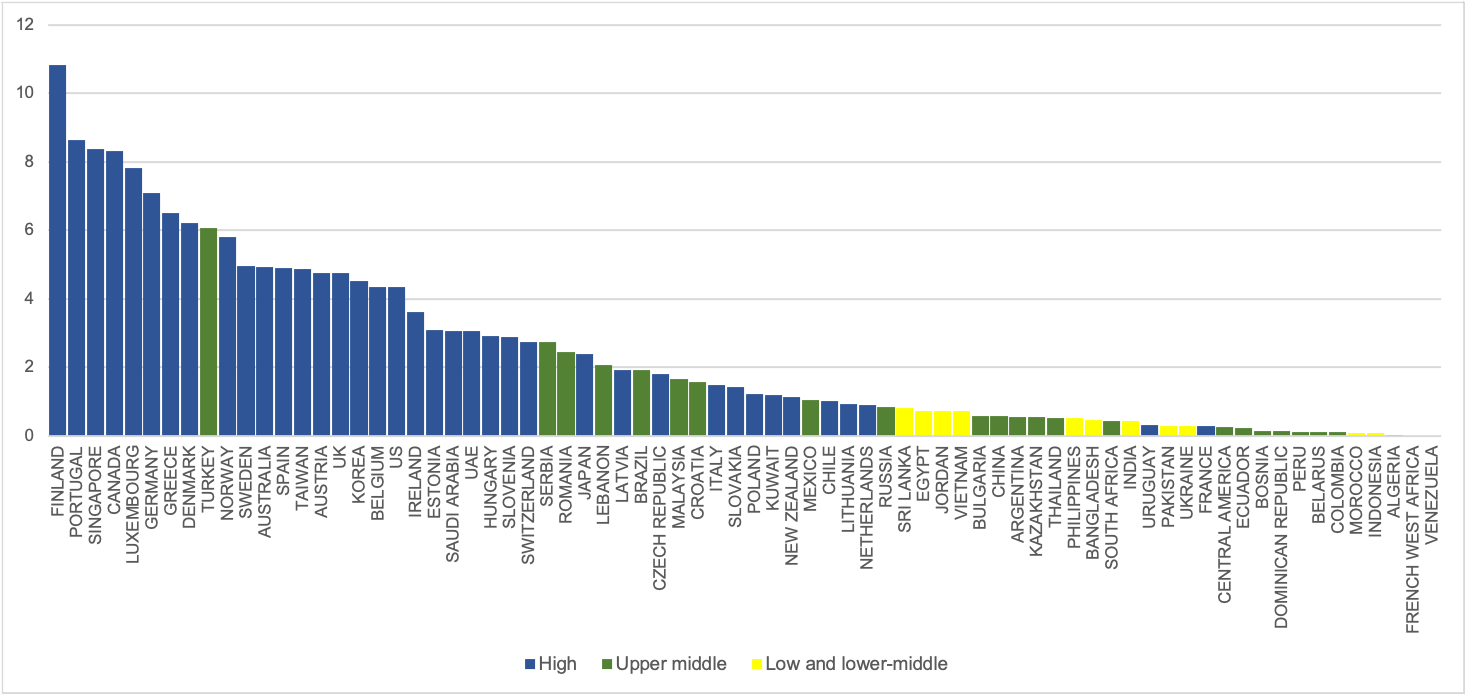

Supplement: S16 Fig — (TIF) [file pgph.0005326.s016.tif]

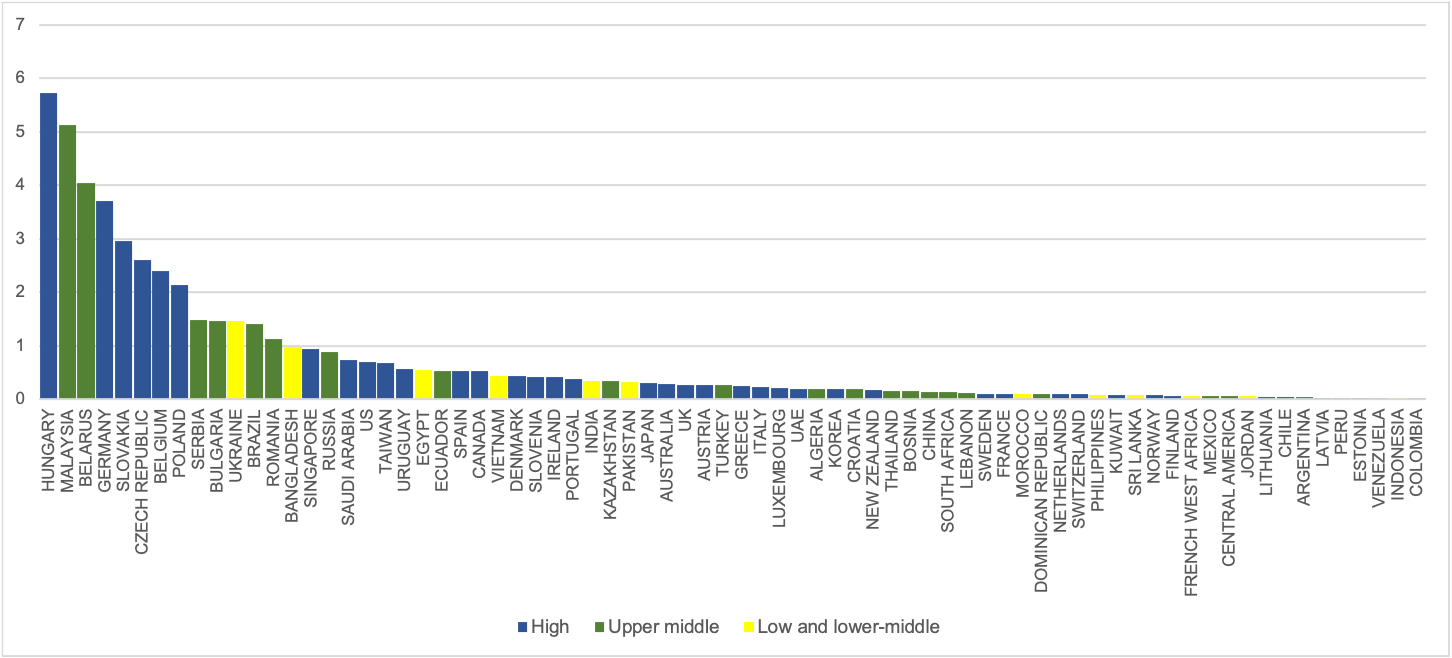

Supplement: S17 Fig — (TIF) [file pgph.0005326.s017.tif]

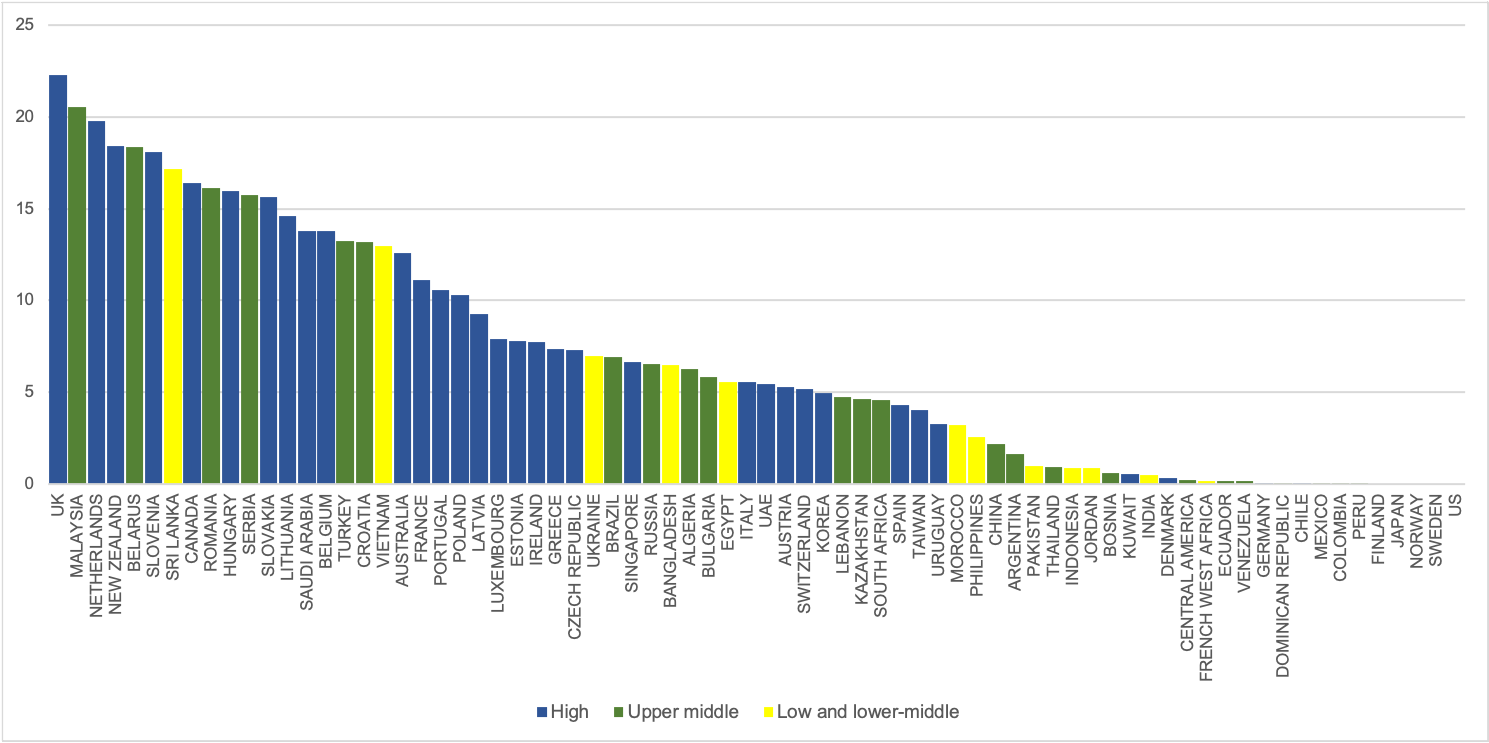

Supplement: S18 Fig — (TIF) [file pgph.0005326.s018.tif]

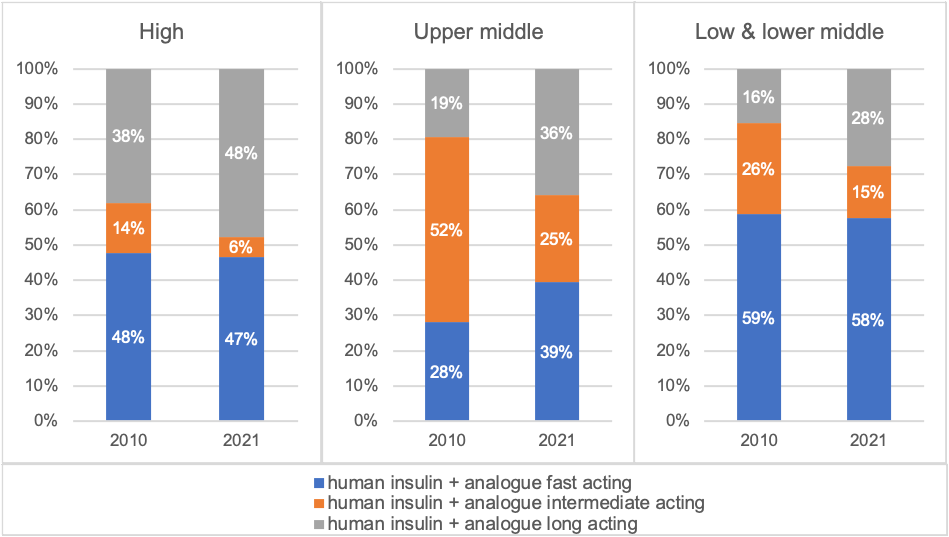

Supplement: S19 Fig — (TIF) [file pgph.0005326.s019.tif]

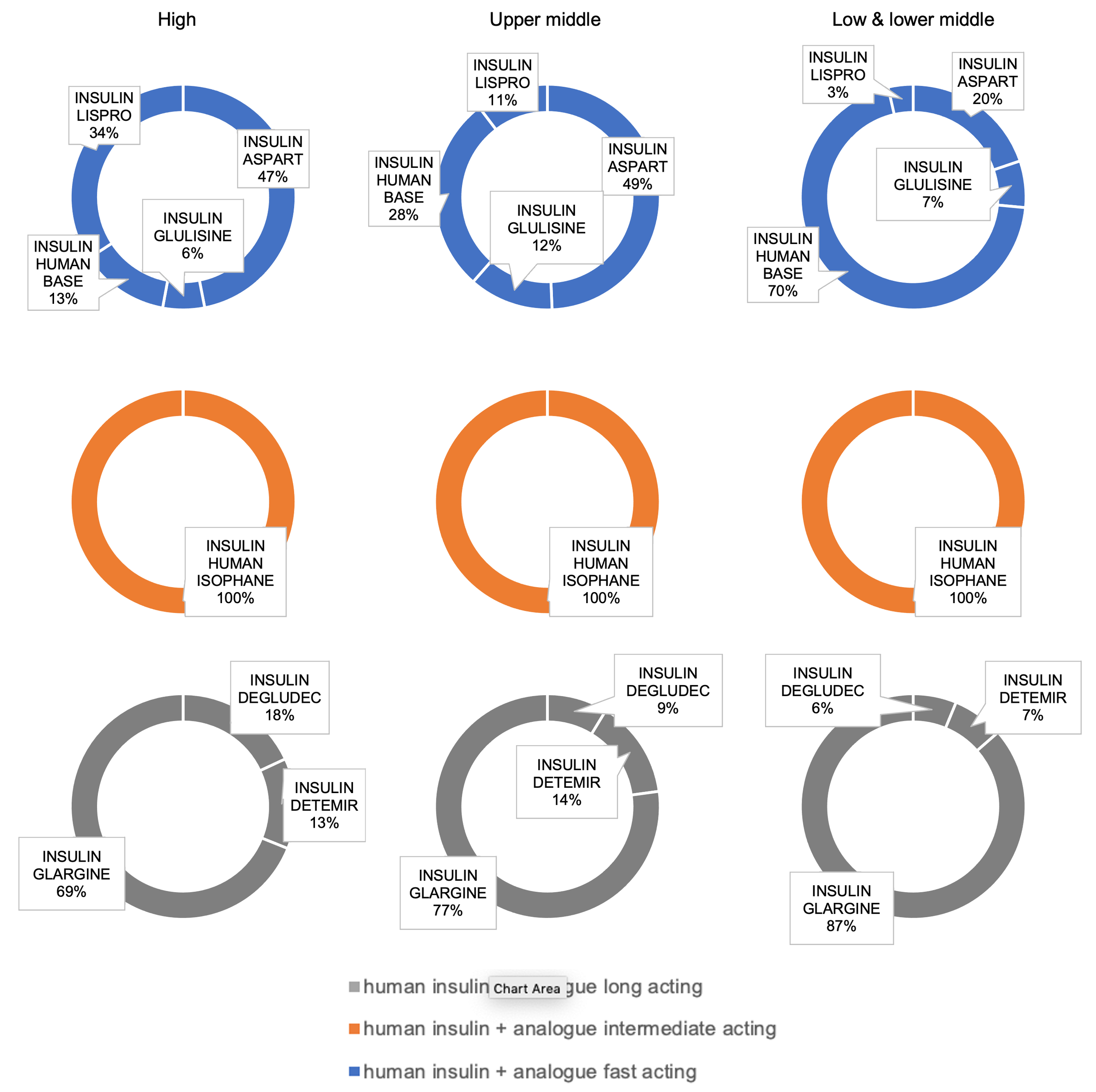

Supplement: S20 Fig — (TIF) [file pgph.0005326.s020.tif]

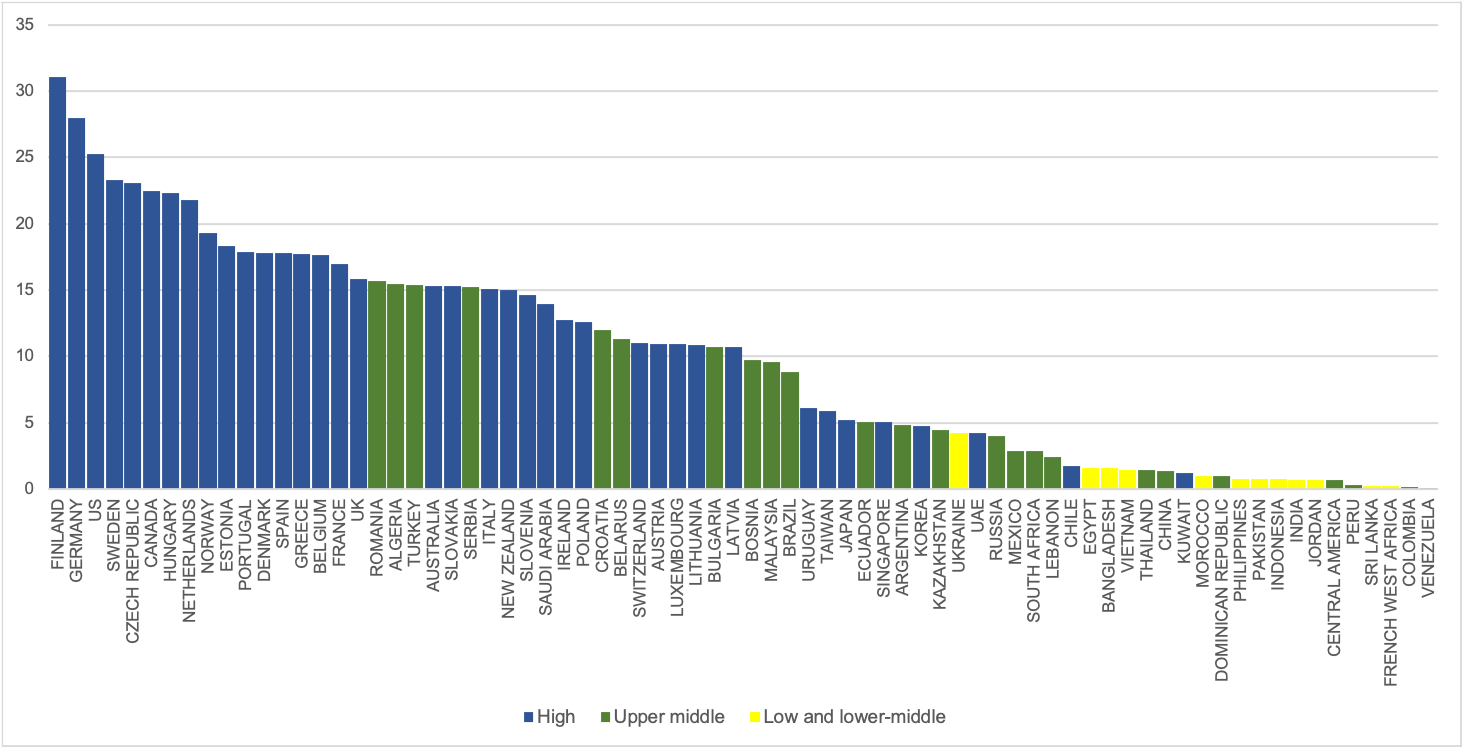

Supplement: S21 Fig — (TIF) [file pgph.0005326.s021.tif]

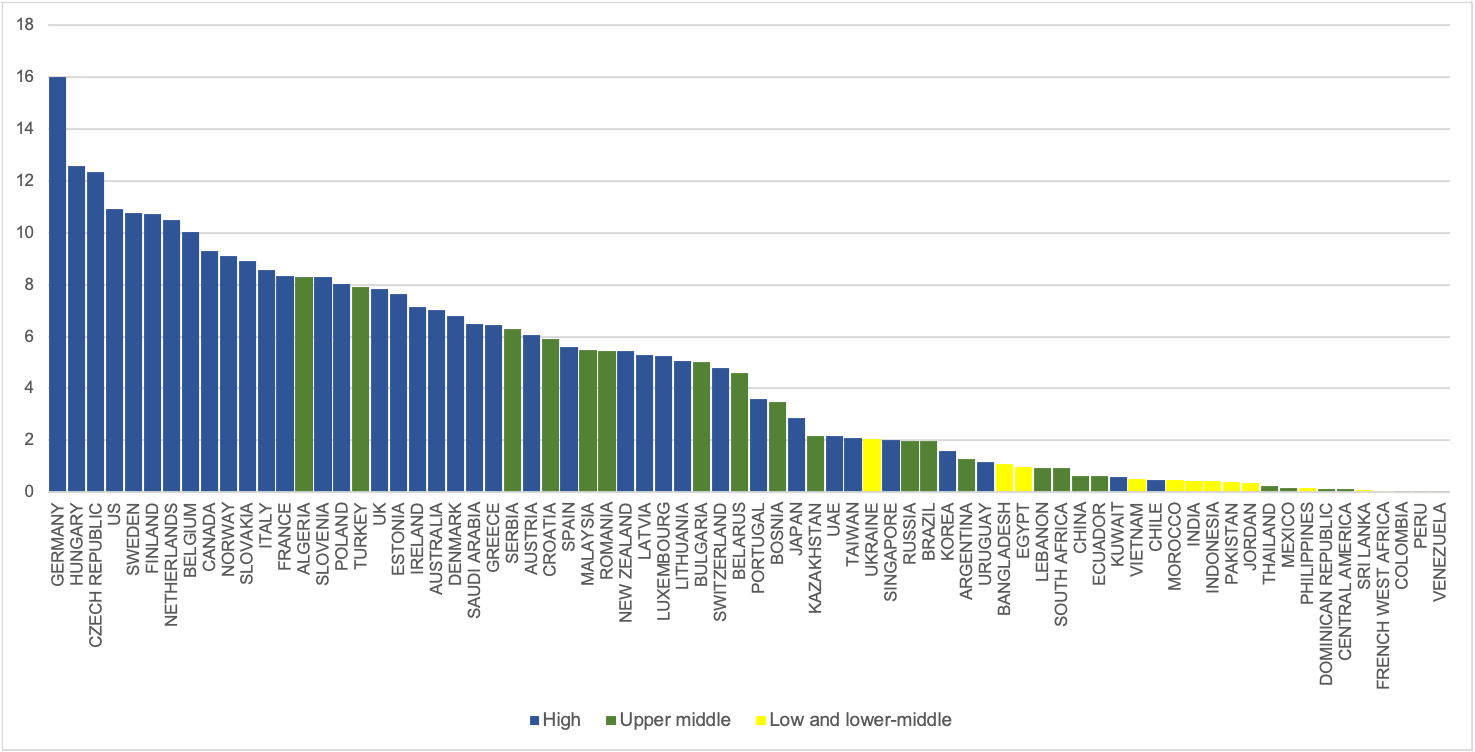

Supplement: S22 Fig — (TIF) [file pgph.0005326.s022.tif]

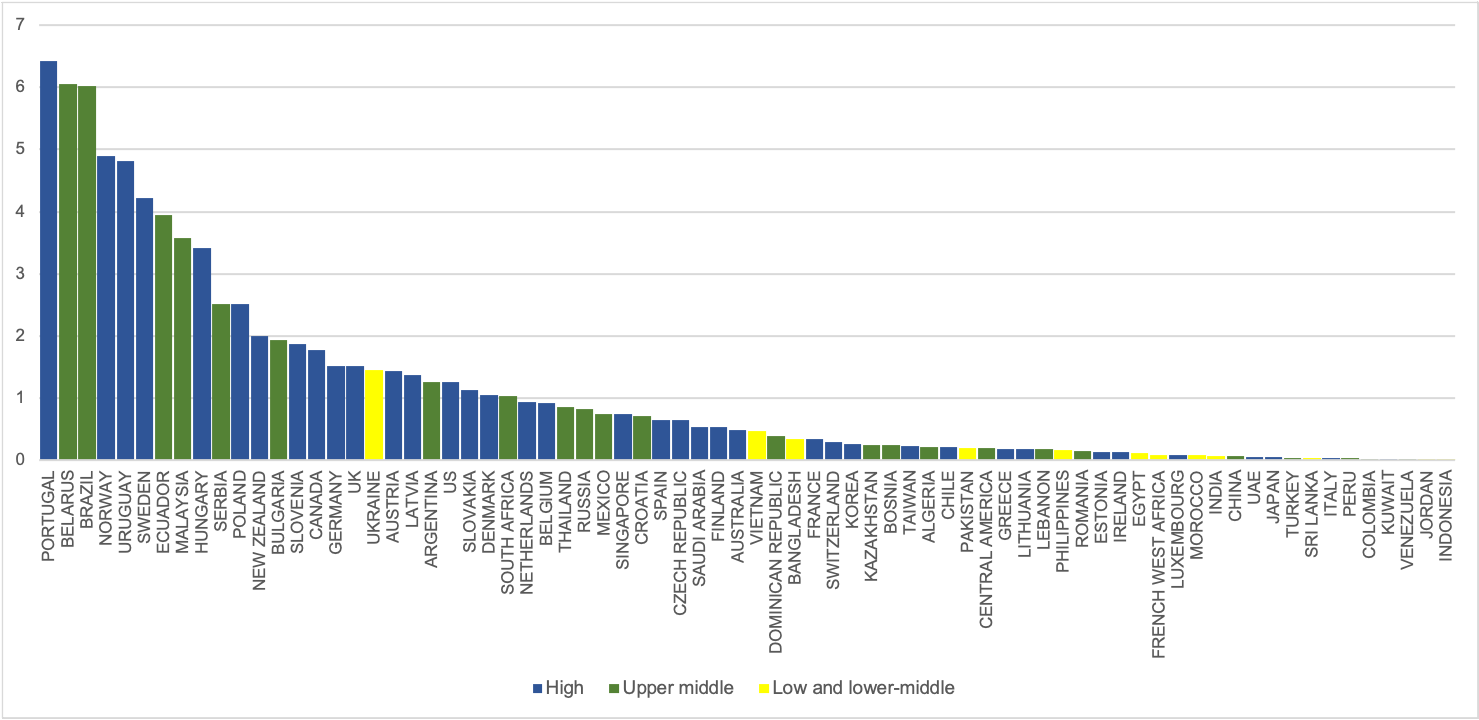

Supplement: S23 Fig — (TIF) [file pgph.0005326.s023.tif]

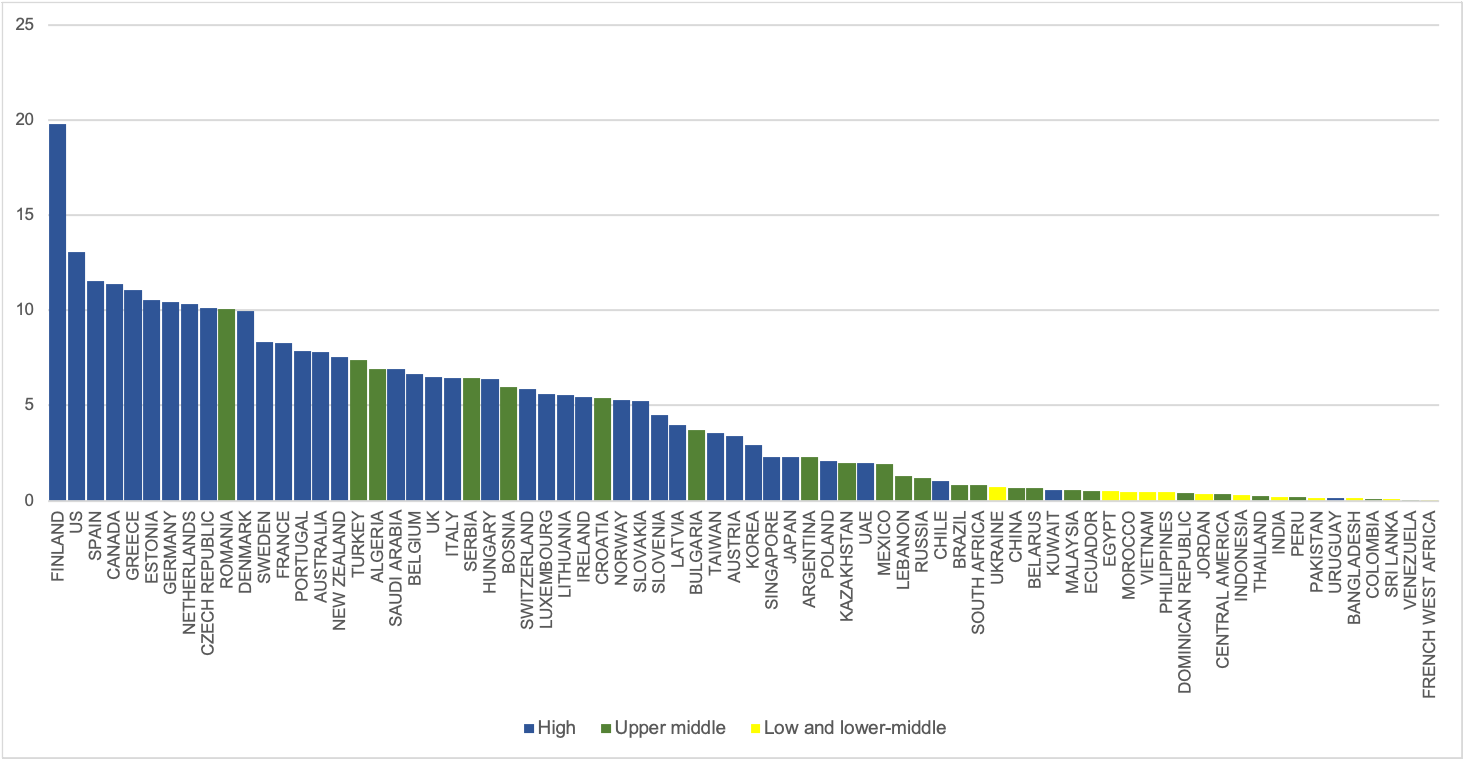

Supplement: S24 Fig — (TIF) [file pgph.0005326.s024.tif]

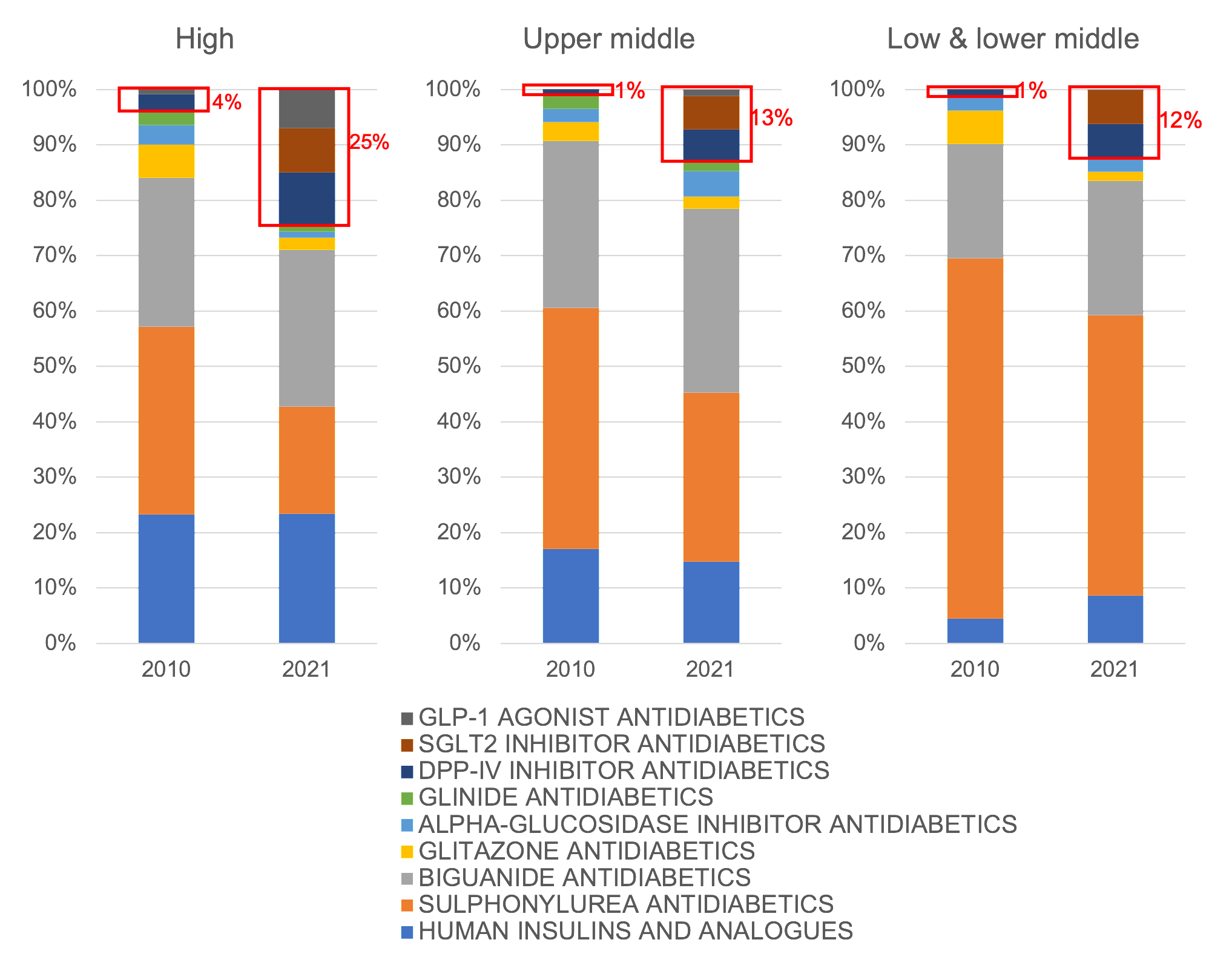

Supplement: S25 Fig — (TIF) [file pgph.0005326.s025.tif]

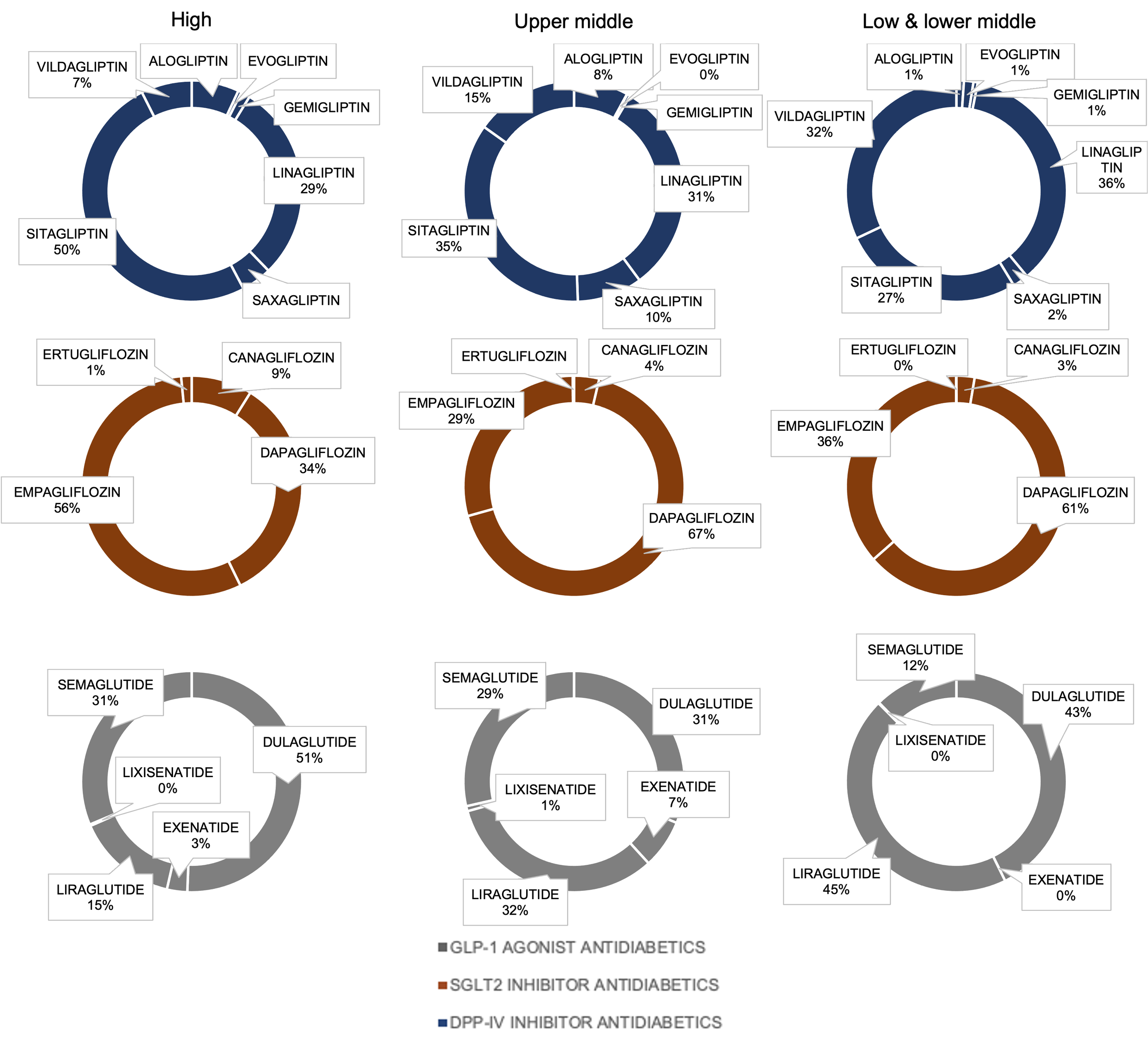

Supplement: S26 Fig — (TIF) [file pgph.0005326.s026.tif]

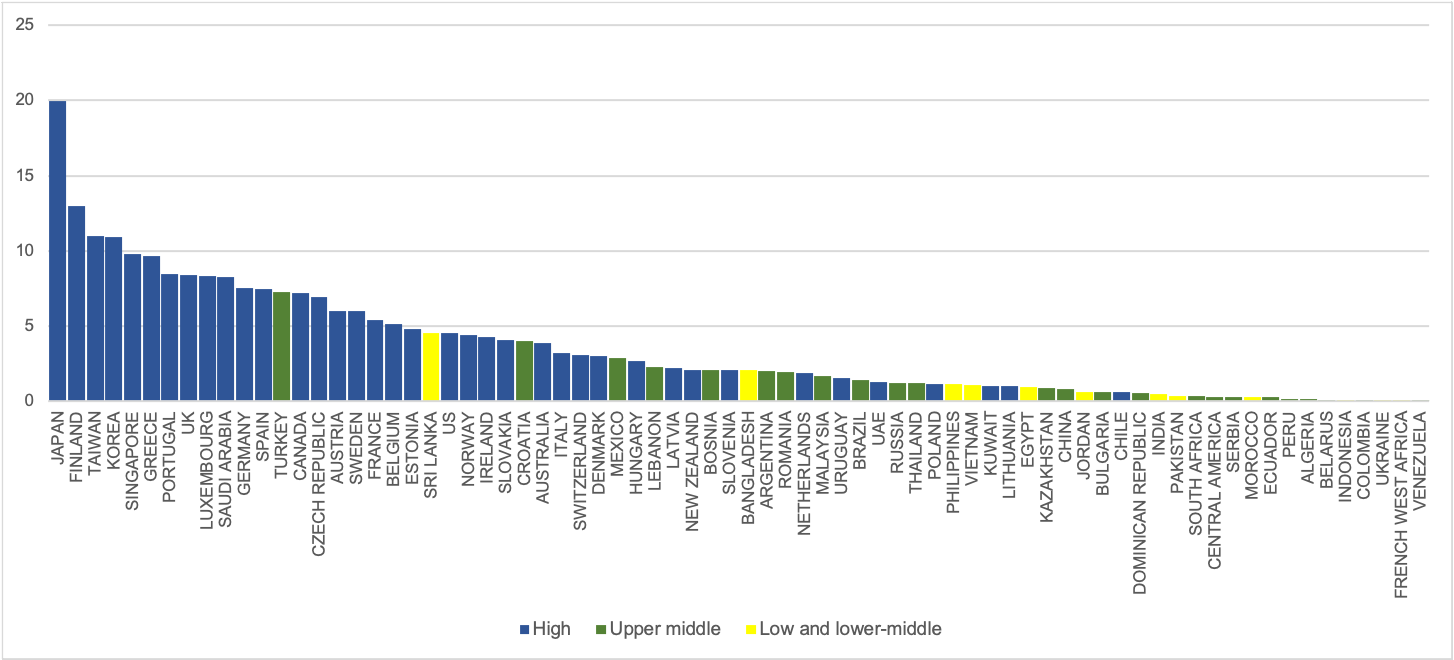

Supplement: S27 Fig — (TIF) [file pgph.0005326.s027.tif]

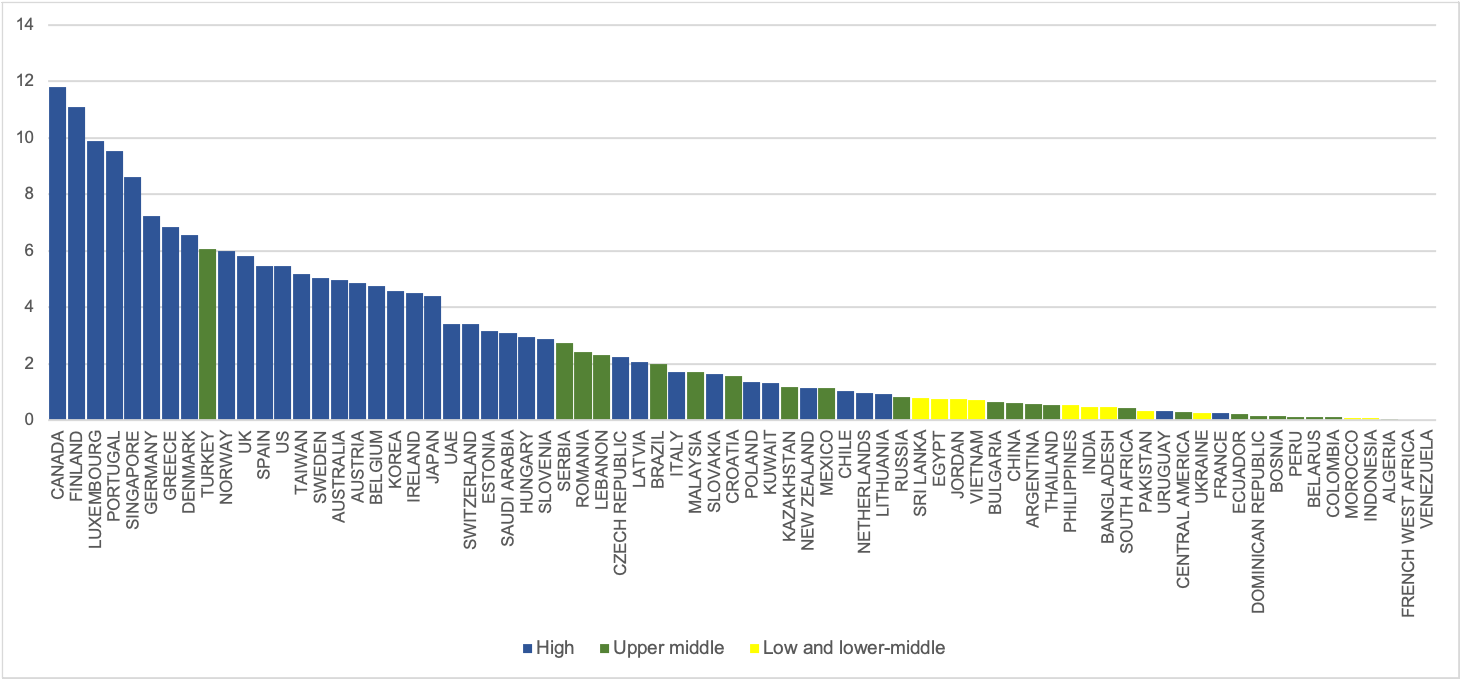

Supplement: S28 Fig — (TIF) [file pgph.0005326.s028.tif]

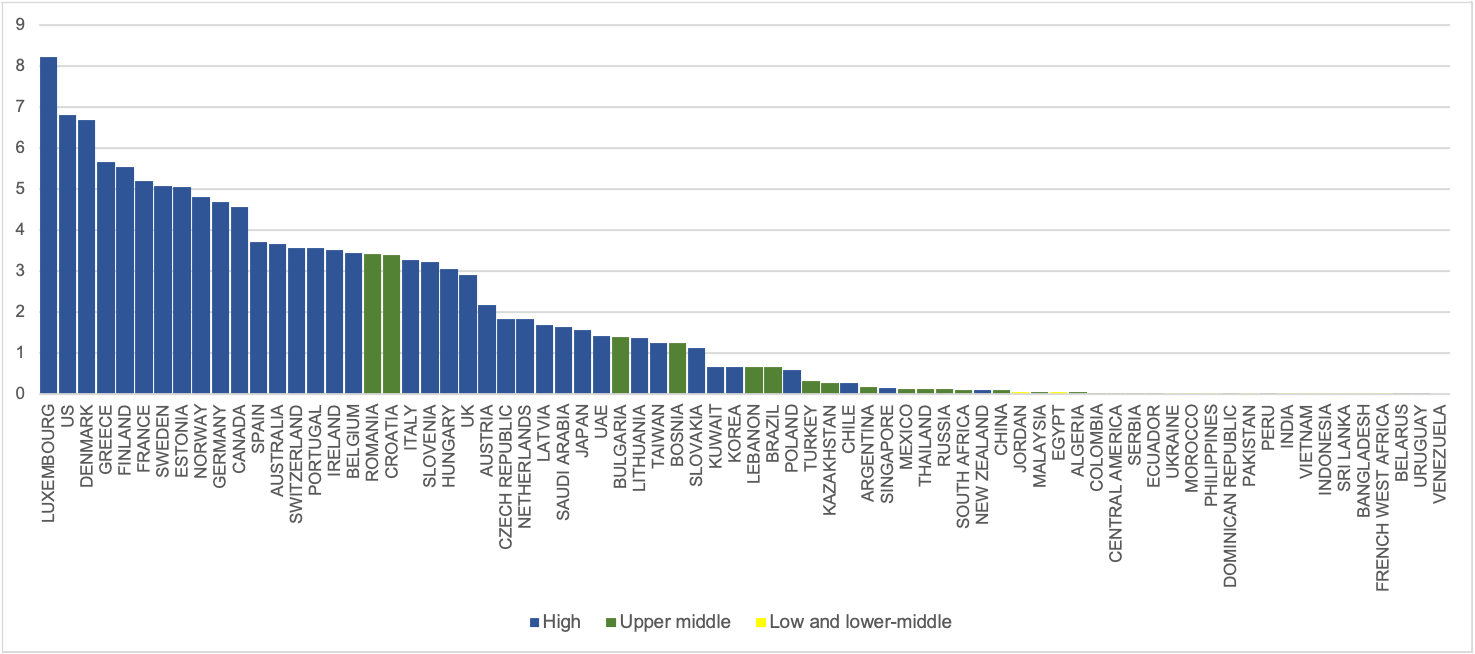

Supplement: S29 Fig — (TIF) [file pgph.0005326.s029.tif]

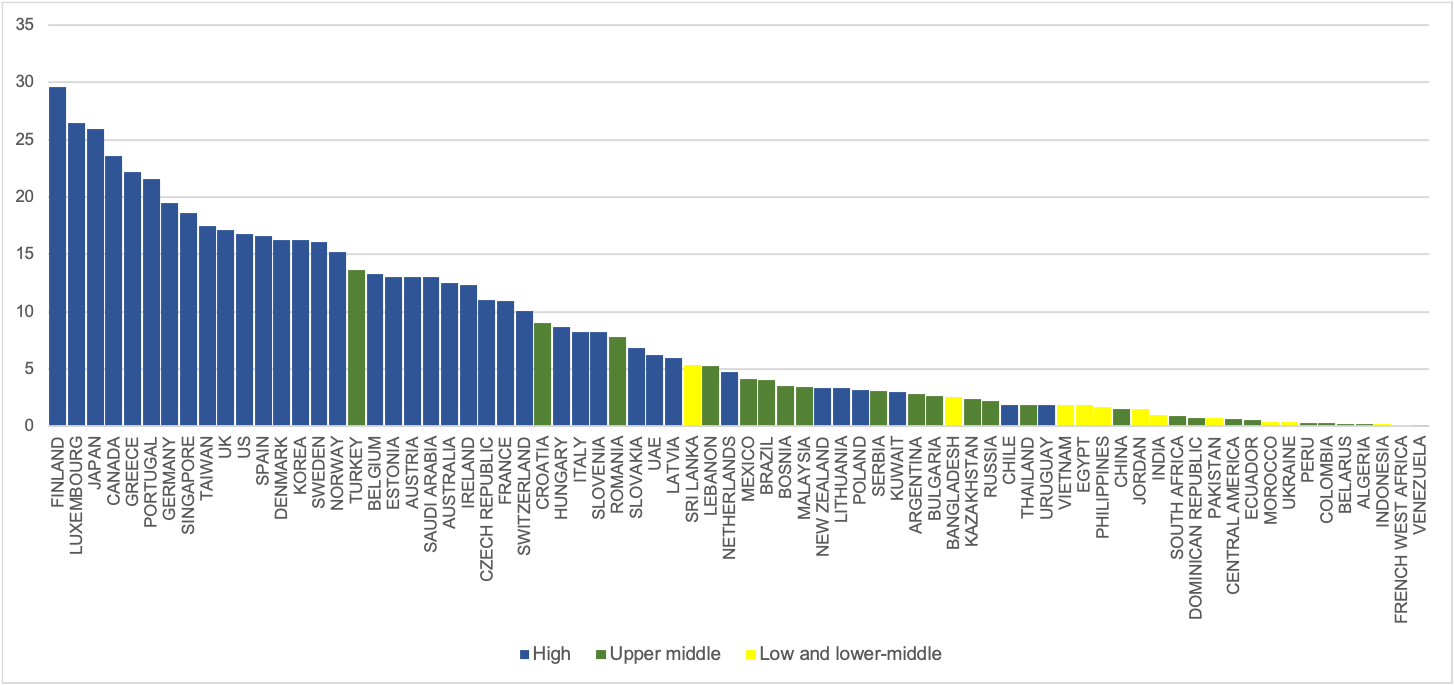

Supplement: S30 Fig — (TIF) [file pgph.0005326.s030.tif]

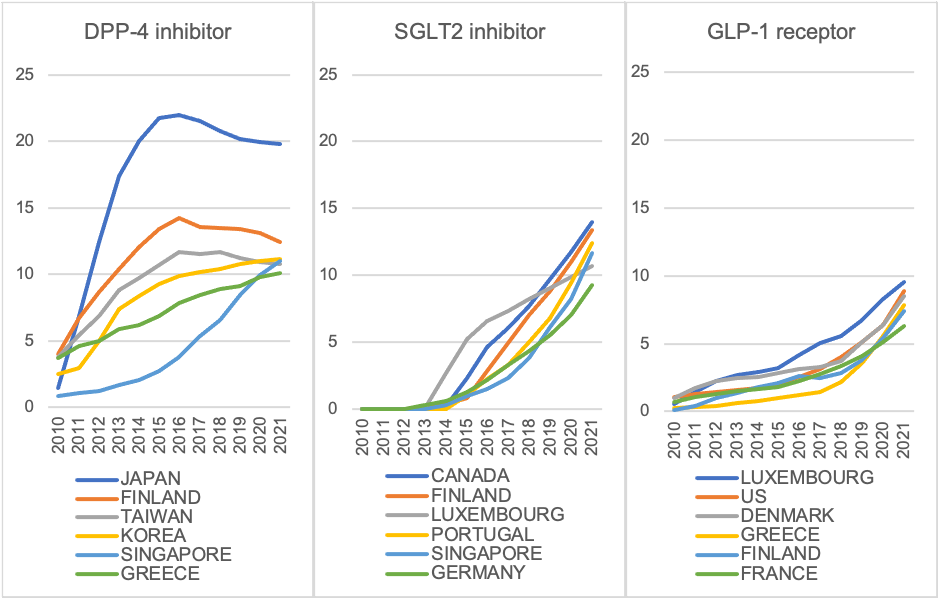

Supplement: S31 Fig — (TIF) [file pgph.0005326.s031.tif]
