# Supplementary material for: Trends in global glucose lowering medication consumption: Insights from pharmaceutical sales data (2010–2021)
Source: PLOS Glob Public Health. 2025 Oct 22;5(10):e0005326. doi: 10.1371/journal.pgph.0005326 (PMC12543110; doi:10.1371/journal.pgph.0005326)
Supplement: S1 Table — (PDF) [file pgph.0005326.s032.pdf]

Country (estimated retail sector market share)	Hospital	Retail
Algeria (80%)	-	2010-2021
Argentina (73%)	-	2010-2021
Australia	2010-2021	2010-2021
Austria	2010-2021	2010-2021
Bangladesh (85%)	-	2010-2021
Belarus	2010-2021	2010-2021
Belgium	2010-2021	2010-2021
Bosnia (65%)	-	2011-2021
Brazil	2010-2021	2010-2021
Bulgaria	2010-2021	2010-2021
Canada	2010-2021	2010-2021
Central America (77%)	-	2010-2021
Chile (71%)	-	2010-2021
China	2010-2021	2010-2021
Colombia (35%)	-	2010-2021
Croatia	2010-2021	2010-2021
Czech Republic	2010-2021	2010-2021
Denmark	2010-2021	2010-2021
Dominican Republic (78%)	-	2010-2021
Ecuador	2010-2021	2010-2021
Egypt (75%)	-	2010-2021
Estonia (88%)	-	2010-2021
Finland	2010-2021	2010-2021
France	2010-2021	2010-2021
French West Africa (86%)	-	2010-2021
Germany	2010-2021	2010-2021
Greece (60%)	-	2010-2021
Hungary	2010-2021	2010-2021
India	2010-2021	2010-2021
Indonesia (26%)	-	2010-2021
Ireland	2010-2021	2010-2021

Italy	2010-2021	2010-2021
Japan	2010-2021	2010-2021
Jordan (71%)	-	2010-2021
Kazakhstan	2010-2021	2010-2021
Korea	2010-2021	2010-2021
Kuwait (35%)	-	2010-2021
Latvia (92%)	-	2010-2021
Lebanon (77%)	-	2010-2021
Lithuania	2010-2021	2010-2021
Luxembourg (98%)	-	2010-2021
Malaysia	2010-2021	2010-2021
Mexico	2010-2021	2010-2021
Morocco (88%)	-	2010-2021
Netherlands	2010-2021	2010-2021
New Zealand	2010-2021	2010-2021
Norway	2010-2021	2010-2021
Pakistan (85%)	-	2010-2021
Peru (67%)	-	2010-2021
Philippines	2010-2021	2010-2021
Poland	2010-2021	2010-2021
Portugal	2010-2021	2010-2021
Romania	2010-2021	2010-2021
Russia	2010-2021	2010-2021
Saudi Arabia	2010-2021	2010-2021
Serbia (65%)	-	2011-2021
Singapore	2010-2021	2010-2021
Slovakia	2010-2021	2010-2021
Slovenia (67%)	-	2010-2021
South Africa	2010-2021	2010-2021
Spain	2010-2021	2010-2021
Sri Lanka (58%)	-	2010-2021
Sweden	2010-2021	2010-2021

Switzerland	2010-2021	2010-2021
Taiwan	2010-2021	2010-2021
Thailand	2010-2021	2010-2021
Turkey	2010-2021	2010-2021
UAE (45%)	-	2010-2021
UK	2010-2021	2010-2021
Ukraine (88%)	-	2010-2021
Uruguay (32%)	-	2010-2021
US	2010-2021	2010-2021
Venezuela (78%)	-	2010-2021
Vietnam	2010-2021	2010-2021
