# Supplementary material for: Trends in global glucose lowering medication consumption: Insights from pharmaceutical sales data (2010–2021)
Source: PLOS Glob Public Health. 2025 Oct 22;5(10):e0005326. doi: 10.1371/journal.pgph.0005326 (PMC12543110; doi:10.1371/journal.pgph.0005326)
Supplement: S2 Table — (PDF) [file pgph.0005326.s033.pdf]

WHO Model List of Essential Medicines-22nd List (2021)	WHO Essential Medicines included in the study
Insulin injection (soluble) including quality-assured biosimilars<br>• Injection: 40 IU/mL in 10 mL vial; 100 IU/mL in 10 mL vial	Insulin injection (soluble) including quality-assured biosimilars<br>• Injection (vial/pen/cartridge/pre-filled syringe): 40 IU/mL; 100 IU/mL
Intermediate-acting insulin including quality-assured biosimilars<br>• Injection: 40 IU/mL in 10 mL vial; 100 IU/mL in 10 mL vial (as compound insulin zinc suspension or isophane insulin)	Intermediate-acting insulin including quality-assured biosimilars<br>• Injection (vial/pen/cartridge/pre-filled syringe): 40 IU/mL; 100 IU/mL
Long-acting insulin analogues including quality-assured biosimilars<br>Therapeutic alternatives: insulin degludec, insulin detemir, insulin glargine<br>• Injection: 100 IU/mL in 3 mL cartridge or pre-filled pen	Long-acting insulin analogues including quality-assured biosimilars<br>Therapeutic alternatives: insulin degludec, insulin detemir, insulin glargine<br>• Injection (vial/pen/cartridge/pre-filled syringe): 100 IU/mL
Empagliflozin<br>Therapeutic alternatives: canagliflozin, dapagliflozin<br>• Tablet: 10 mg; 25 mg	Empagliflozin<br>Therapeutic alternatives: canagliflozin, dapagliflozin<br>• Tablet/cap: 10 mg; 25 mg
Gliclazide<br>Therapeutic alternatives: 4th level ATC chemical subgroup (A10BB Sulfonylureas)<br>• Solid oral dosage form: (controlled-release tablets): 30 mg; 60 mg; 80 mg	Gliclazide<br>Therapeutic alternatives: 4th level ATC chemical subgroup (A10BB Sulfonylureas)<br>• Tablet/cap: 30 mg; 60 mg; 80 mg
Metformin<br>• Tablet: 500 mg (hydrochloride)	Metformin

- Tablet/cap: 500 mg (hydrochloride)
