# Supplementary material for: Trends in global glucose lowering medication consumption: Insights from pharmaceutical sales data (2010–2021)
Source: PLOS Glob Public Health. 2025 Oct 22;5(10):e0005326. doi: 10.1371/journal.pgph.0005326 (PMC12543110; doi:10.1371/journal.pgph.0005326)
Supplement: S3 Table — (PDF) [file pgph.0005326.s034.pdf]

	High	Upper middle	Low & lower middle
**Insulin**			
Fast-acting insulin	2010: 4.68<br>2021: 6.98	2010: 0.47<br>2021: 1.63	2010: 0.09<br>2021: 0.46
Intermediate-acting insulin	2010: 1.37<br>2021: 0.67	2010: 0.55<br>2021: 0.54	2010: 0.06<br>2021: 0.11
Long-acting insulin	2010: 3.40<br>2021: 6.29	2010: 0.31<br>2021: 0.94	2010: 0.03<br>2021: 0.33
**New drug classes**			
DPP-4 inhibitors	2010: 1.12<br>2021: 4.59	2010: 0.16<br>2021: 1.12	2010: 0.02<br>2021: 0.59
SGLT2 inhibitors	2010: 0.00<br>2021: 5.38	2010: 0.00<br>2021: 0.74	2010: 0.00<br>2021: 0.65
GLP-1 receptor agonists	2010: 0.08<br>2021: 4.02	2010: 0.00<br>2021: 0.16	2010: 0.00<br>2021: 0.01
