# Supplementary material for: Trends in global glucose lowering medication consumption: Insights from pharmaceutical sales data (2010–2021)
Source: PLOS Glob Public Health. 2025 Oct 22;5(10):e0005326. doi: 10.1371/journal.pgph.0005326 (PMC12543110; doi:10.1371/journal.pgph.0005326)
Supplement: S4 Table — DDD/TID: defined daily doses per thousand inhabitants per day; DALY: disability-adjusted life year. (PDF) [file pgph.0005326.s035.pdf]

	Median diabetes prevalence rate in 2021 (per 100,000)	Median diabetes age-standardised DALY rate in 2021 (per 100,000)	Median diabetes death rate in 2021 (per 100,000)	Median consumption rate of antidiabetic medications in 2021 (DDD/TID)
High	5,232	578	9.35	67
Upper middle	6,231	1,047	20.80	33
Low & lower middle	6,452	1,118	38.85	15
