# Supplementary material for: Trends in global glucose lowering medication consumption: Insights from pharmaceutical sales data (2010–2021)
Source: PLOS Glob Public Health. 2025 Oct 22;5(10):e0005326. doi: 10.1371/journal.pgph.0005326 (PMC12543110; doi:10.1371/journal.pgph.0005326)
Supplement: S5 Table — DALY: disability-adjusted life year. (PDF) [file pgph.0005326.s036.pdf]

	Median percentage change in diabetes prevalence rate, 1990-2021 (%)	Median percentage change in diabetes age-standardised DALY rate, 1990-2021 (%)	Median percentage change in diabetes death rate, 1990-2021 (%)	Median percentage change in consumption rate of antidiabetic medications, 2010-2021 (%)
High	90%	21%	-30%	31%
Upper middle	87%	32%	2%	91%
Low & lower middle	100%	46%	35%	115%
